# Genome-wide association between single nucleotide polymorphisms with beef fatty acid profile in Nellore cattle using the single step procedure

**DOI:** 10.1186/s12864-016-2511-y

**Published:** 2016-03-09

**Authors:** Marcos V. A. Lemos, Hermenegildo Lucas Justino Chiaia, Mariana Piatto Berton, Fabieli L. B. Feitosa, Carolyn Aboujaoud, Gregório M. F. Camargo, Angélica S. C. Pereira, Lucia G. Albuquerque, Adrielle M. Ferrinho, Lenise F. Mueller, Monica R. Mazalli, Joyce J. M. Furlan, Roberto Carvalheiro, Daniel M. Gordo, Rafael Tonussi, Rafael Espigolan, Rafael Medeiros de Oliveira Silva, Henrique Nunes de Oliveira, Susan Duckett, Ignacio Aguilar, Fernando Baldi

**Affiliations:** Departamento de Zootecnia, Faculdade de Ciências Agrárias e Veterinárias, Universidade Estadual Paulista, Via de acesso Prof. Paulo Donato Castellane, s/no, CEP 14884-900 Jaboticabal, São Paulo Brazil; Departamento de Nutrição e Produção Animal, Faculdade de Medicina Veterinária e Zootecnia, Universidade de São Paulo, Avenida Duque de Caxias Norte, 225, CEP 13635-900 Pirassununga, São Paulo Brazil; Department of Animal and Veterinary Science, Clemson University, Clemson, SC USA; Department of Animal Breeding Montevideo, National Institute of Agricultural Research of Uruguayy, Montevideo, Uruguay

**Keywords:** *Bos indicus*, Fatty acid composition, Heritability, Genetic markers, Mapping, ssGWAS

## Abstract

**Background:**

Saturated fatty acids can be detrimental to human health and have received considerable attention in recent years. Several studies using taurine breeds showed the existence of genetic variability and thus the possibility of genetic improvement of the fatty acid profile in beef. This study identified the regions of the genome associated with saturated, mono- and polyunsaturated fatty acids, and n-6 to n-3 ratios in the *Longissimus thoracis* of Nellore finished in feedlot, using the single-step method.

**Results:**

The results showed that 115 windows explain more than 1 % of the additive genetic variance for the 22 studied fatty acids. Thirty-one genomic regions that explain more than 1 % of the additive genetic variance were observed for total saturated fatty acids, C12:0, C14:0, C16:0 and C18:0. Nineteen genomic regions, distributed in sixteen different chromosomes accounted for more than 1 % of the additive genetic variance for the monounsaturated fatty acids, such as the sum of monounsaturated fatty acids, C14:1 cis-9, C18:1 trans-11, C18:1 cis-9, and C18:1 trans-9. Forty genomic regions explained more than 1 % of the additive variance for the polyunsaturated fatty acids group, which are related to the total polyunsaturated fatty acids, C20:4 n-6, C18:2 cis-9 cis12 n-6, C18:3 n-3, C18:3 n-6, C22:6 n-3 and C20:3 n-6 cis-8 cis-11 cis-14. Twenty-one genomic regions accounted for more than 1 % of the genetic variance for the group of omega-3, omega-6 and the n-6:n-3 ratio.

**Conclusions:**

The identification of such regions and the respective candidate genes, such as *ELOVL5*, *ESSRG*, *PCYT1A* and genes of the *ABC* group (*ABC5*, *ABC6* and *ABC10*), should contribute to form a genetic basis of the fatty acid profile of Nellore (*Bos indicus*) beef, contributing to better selection of the traits associated with improving human health.

**Electronic supplementary material:**

The online version of this article (doi:10.1186/s12864-016-2511-y) contains supplementary material, which is available to authorized users.

## Background

High consumption of saturated fatty acids (SFA) is associated with increased serum levels of cholesterol and low-density lipoproteins (LDL), considered risk factors for the occurrence of cardiovascular disease [1]. The SFAs prevalent in beef fat are the myristic (C14:0), palmitic (C16:0) and stearic (C18:0) fatty acids [2, 3]. The polyunsaturated fatty acids (PUFA) present in beef, such as linoleic (C18:2 n-6) and linolenic (C18:3 n-3), and monounsaturated (MUFA), as oleic acid (C18:1 n-9) protect the cardiovascular system, since moderate consumption has been linked to decreasing serum cholesterol and increasing high-density lipoprotein (HDL) [4–6].

Furthermore, the fat of ruminants is a natural source of conjugated isomers of linoleic acid (CLA c9 t11), such as C18:2 cis-9 trans-11 [7], which are synthesized in the rumen as a result of biohydrogenation of fatty acids, performed by microorganisms [8]. The CLAs have a positive effect on human health, related to anticancer activity, immune functions, and potential beneficial effects on coronary heart disease [9, 10]. Strategies such as diet [11] and gene manipulation [12] have been used to satisfy the growing consumer demand for protein sources with healthier lipid profile. Thus, regions associated with the expression of beef fatty acid profile have been identified aiming to locate key genes in the genome [13–15] that contribute to these features. This genomic tool will assist the use of information that is beneficial to human health.

Recently, several genome-wide association studies using taurine breeds and their crosses [16–19] have identified genetic variants for fatty acid (FA) profile in beef. These results would allow producers to select for desirable nutritional values with respect to meat FA that could increase beef value and consumer satisfaction. However, there are limited number of genomic association studies with a large sample size that aim to determine genome regions associated with the meat fatty acid profile of zebu cattle reared in tropical conditions [3]. The study of [3] utilized 386 Nellore steers, sired from 34 unrelated sires, from an experimental herd, and applied the Bayes B method to perform the genome-wide association analysis. In the literature, there is some controversy regarding the capacity of different methods to identify genomic regions related to phenotypes due to differences in method presuppositions [20–22]. Moreover, it is important to perform genome-wide association studies (GWAS) in indicine populations due to differences in environment and management conditions, and also differences in allele frequency of genetic markers and QTL, that would influence the results. The identification of genomic regions that affect the meat fatty acid composition may become an important and highly applicable tool to improve the nutritional value of zebu meat given the expensive and difficult nature of collecting phenotypic records.

The objective of this study was to identify regions associated with saturated, mono- and polyunsaturated and n-6 to n-3 ratios, in the *Longissimus* thoracis muscle from confined Nellore, using the single-step method.

## Results and discussion

### Fatty acid profile

The individual fatty acids with the highest concentration in the intramuscular fat of *Longissimus thoracis* were C16:0, C18:1 cis-9, C18:1 trans-11, and C18:0, representing 67.3 % of its fat composition (Table 1). These results agreed with those reported by some authors [2, 13, 23, 24] who observed high levels of palmitic, stearic and oleic FAs. Some authors [2, 3] also reported that palmitic fatty acid was the predominant FA in beef fat. In Nellore finished in feedlot [13], oleic acid (37.46 %) displayed the highest concentration in intramuscular fat. The myristic and palmitic FAs are associated with an increase in circulating LDL cholesterol due to interference with hepatic LDL receptors [25]. The saturated fatty acid were predominant, followed by the MUFAs and PUFAs. Similar results [23] were reported for Nellore cattle, 43.93 % (SFA), 42.33 % (MUFA) and 12.8 % (PUFA). However, studies using taurine [26] and Nellore [13] breeds found similar concentrations for SFA and MUFA, 47 and 47.5; and 47.23, and 48.34 %, respectively.

In the present study, the n-6:n-3 ratio was less than 4:1, the value recommended by the Department of Health and some authors [27, 28]. Excessive amounts of n-6 and a high n-6:n-3 ratio can lead to pathogenies, including cardiovascular, inflammatory, cancer and autoimmune diseases while increased levels of omega-3 fatty acids help to suppress such effects [29]. Studies have associated a 4:1 ratio to 70 % decrease in mortality of humans, and also to preventing cardiovascular diseases [30]. The Department of Health recommends values above 0.45 for the PUFA/SFA ratio. The average value for this ratio in this study is below this limit (0.35). A PUFA/SFA ratio of 0.11 has been reported in beef purchased at supermarkets in the UK [31]. However, this PUFA/SFA ratio may vary depending upon genetic and dietary factors [12].

### Heritability estimates

The Gibbs sampling approach was used to estimate de (co)variance components and the convergence for all estimated parameters was verified through inspection of trace-plots and the Geweke’s [32] and Heidelberger and Welch convergence diagnostic [33] indicated convergence of the chain. Thus, the burn-in period considered was sufficient to reach the convergence in all parameter estimates. The posterior marginal distributions of heritability estimates for fatty acid profile, presented showed accurate values, tending to normal distribution (Table 2). The symmetric distributions of central tendency statistics are indicative that the analyses are reliable.

**Table 1:** Descriptive statistics for the fatty acids profile of Nellore beef^a^

Trait	Nomenclature	Mean	^b^SD	^c^N
Intramuscular fat	IMF (in percentage)	0.83	0.42	934
Lauric	C12:0	0.06	0.18	538
Myristic	C14:0	2.13	0.54	867
Myristoleic	C14:1	0.32	0.22	824
Palmitic	C16:0	21.03	2.49	867
Palmitoleic	C16:1	2.18	0.78	937
Stearic	C18:0	13.63	3.32	783
Elaidic	C18:1n9t	2.91	5.07	483
Oleic	C18:1n9c	30.64	4.98	934
Vaccenic (TVA)	C18:1 t11	1.29	0.01	878
Linoleic (LA)	C18:2n6	8.32	3.63	865
Linolenic	C18:3n3	0.59	0.26	858
CLA-*cis*	C18:2c9t11	0.26	0.16	727
CLA-*trans*	C18:2 trans10 cis 12	0.20	0.12	241
Docosahexaenoic (DHA)	C22:6 n3	0.95	0.39	865
Eicosatrienoic	C20:3 n6 cis-8,11,14	0.49	0.19	862
Sum of SFA		40.66	6.12	868
Sum of MUFA		37.55	8.05	868
Sum of PUFA		13.42	5.57	868
Sum of Omega-3		3.81	1.55	868
Sum of Omega-6		9.35	4.44	868
n6/n3 ratio		2.54	0.97	868
PUFA/SFA ratio		0.35	0.20	868

^a^The concentration of fatty acids are expressed as a percentage of total fatty acid methyl esters (FAME) quantified ^b^ *SD* standard deviation ^c^ *N* number of animals with records

**Table 2:** Descriptive statistics and highest posterior density (HPD) region for fatty acid heritability estimates

Trait	Nomenclature	Mean	Median	SD^a^	HPD	
					Low limit	Upper limit
Lauric	C12:0	0.64	0.69	0.24	0.17	0.99
Myristic	C14:0	0.27	0.26	0.10	0.04	0.46
Palmitic	C16:0	0.20	0.19	0.09	0.03	0.38
Stearic	C18:0	0.12	0.11	0.09	0.001	0.30
Myristoleic	C14:1	0.17	0.28	0.09	0.0003	0.62
Palmitoleic	C16:1	0.08	0.07	0.06	0.0002	0.21
Elaidic	C18:1 n9t	0.24	0.21	0.16	0.006	0.57
Oleic	C18:1 n9c	0.28	0.16	0.10	0.08	0.48
Vaccenic (TVA)	C18:1 trans-11	0.11	0.14	0.06	0.003	0.24
Linoleic (LA)	C18:2 n6	0.18	0.17	0.08	0.04	0.34
Linolenic acid (LA)	C18:3 n3	0.15	0.15	0.08	0.02	0.31
Eicosatrienoic	C20:3 n6 cis-8,11,14	0.14	0.13	0.09	0.02	0.31
Docosahexaenoic acid (DHA)	C22:6 n3	0.15	0.14	0.10	0.01	0.32
CLA	Cis9 Trans-11	0.09	0.08	0.06	0.004	0.21
	Trans10 Cis-12	0.52	0.54	0.27	0.02	0.94
Arachidonic acid	C20:4 n-6	0.14	0.11	0.11	0.0007	0.38
Total of SFA		0.12	0.12	0.07	0.0001	0.31
Total of MUFA		0.20	0.12	0.15	0.0005	0.31
Total of PUFA		0.08	0.11	0.05	0.0009	0.32
Omega-3		0.11	0.09	0.07	0.00008	0.29
Omega-6		0.23	0.11	0.10	0.0001	0.31
Omega-9		0.20	0.17	0.14	0.0002	0.46
Omega-6/omega-3 ratio		0.11	0.09	0.08	0.002	0.70
PUFA/SFA ratio		0.07	0.06	0.05	0.0001	0.17

^a^ *SD* standard deviation

The heritability estimates for the individual fatty acids profile of intramuscular fat in the *Longissimus thoracis* muscle were mostly moderate, but low for the C18:0, C16:1 and CLA cis-9 trans-11 acids and high for the C12:0 and CLA trans-10 cis-12 acids (Table 2). The linolenic FA heritability estimate obtained in this study was similar to that found by some authors (0.13) [13] and lower than that reported by other studies (0.58) [34]. However, higher estimates have been reported for linolenic acid in other studies (0.21) [35], and also for palmitoleic acid (0.15) [13] and (0.49) [16]. Higher heritability estimates were reported for linoleic FA, 0.34 and 0.58, respectively, in the intramuscular fat of Japanese Black cattle, suggesting that genetic influence on linoleic acid varies among breeds [34, 36]. Lower and similar heritability was estimated for myristic (0.18) and palmitic (0.21) FAs [26], respectively, while studies with Nellore estimated low heritability for these FAs, ranging from 0.08 to 0.17 [13]. However, studies [37] reported high estimates for the myristoleic FA (0.51) and [34] also found high estimates for myristic (0.70), palmitic (0.65), myristoleic (0.60) and linoleic (0:58). Other authors also estimated low heritability estimates for the stearic [26, 37], CLA cis-9 trans-11 [13, 37] and arachidonic [13] FAs.

The heritability estimates for the sum of omega-3 series fatty acids, the n-6:n-3 ratio, and the sum of saturated and polyunsaturated fatty acids and their ratios were low (<0.12). However, moderate heritability estimates were obtained for the sum of monounsaturated fatty acids and the sum of n-6 and n-9 fatty acids. Studies estimated low heritability estimates for SFA and MUFA and moderate values for n-3 and n-6 [13, 37]. Low to moderate heritability estimates for PUFA (0.05 to 0.12), MUFA (0.06 to 0.20) and SFA (0.07 to 0.30) have been reported [26, 37, 38]. Nevertheless, other studies reported higher heritability estimates for these groups of fatty acids, 0.47 for PUFA, 0.35 to 0.66 for SFA and 0.35 to 0.68 for MUFA in Japanese Black cattle [31, 36]. Recently, authors also estimated high heritability for SFA (0.54) and MUFA (0.54) and, therefore, concluded that there is sufficient genetic variation in the fatty acid profile of cattle subcutaneous fat to respond to selection [24]. The results of this study suggest that it is possible to change the lipids composition of intramuscular fat of Nellore meat through selection. This information is important for breeding programs of zebu breeds that aim at improving the beef fatty acid composition.

### GWAS and genomic regions

The windows of 10 continuous SNPs that accounted for more than 1 % of the genetic variance were used to search for putative candidate genes (PCG), which are represented in Tables 3, 4, 5 and 6. The results indicated a total of 115 different windows that explained more than 1 % of the genetic variance for the 22 fatty acids studied.

**Table 3:** Genomic regions associated with the saturated fatty acids profile in intramuscular fat of the *Longissimus thoracis* muscle of Nellore

Traits	QTL window	% Variance explained SNP window ^a^	PCG^b^
Total of SFA	1:113607033-113638343	1.29	-^c^
	4:8338571-8365876	6.64	CDK14
	5:115737350-115767786	2.59	-
	23:51299188-51326222	3.71	GMDS
C12:0	7:10826539-10852759	1.25	-
	16:20584009-20601201	1.46	ESRRG
	29:35323539-35335022	1.02	-
C14:0	1:71431023-71499402	2.52	SLC51A PCYT1A TCTEX1D2
	1:109079423-112985104	1.43	PLCH1
	2:12006597-12035487	2.84	-
	2:95199347-95226934	2.84	ADAM23
	2:108339996-108355638	3.93	-
	8:4017767-4040618	1.61	GALNTL6
	9:5964257-5996599	1.34	-
	9:75828407-75861439	1.34	PEX7
	12:1848851-1867387	1.23	-
	17:33139235-33161695	1.38	-
	29:14239868-14284421	1.25	-
C16:0	1:71431023-71499402	2.48	SLC51A PCYT1A TCTEX1D2
	2:12009907-12039794	1.95	-
	3:6449321-7462185	1.63	UAP UHMK1 HSD17B7
	4:27962298-27989717	1.76	-
	8: 4017767-4040618	2.47	GALNTL6
	9:7517866-7545663	1.73	BAI3
	12:21267027-22190566	2.90	ATP7B DHRS12
	20:1657499-1668052	1.80	FAM196B DOCK2
C18:0	1:96012245-96039235	6.14	FNDC3B
	2:125065946-125101312	1.45	EPB41
	3:115395556-115435819	8.33	-
	8:100281732-100312597	3.90	TMEM245 MIR32
	17:66287675-66303849	1.45	SVOP

^a^Window that consists of continuous 10 SNPs

^b^Positional/putative candidate gene

^c^No PCG associated with the trait

**Table 4:** Genomic regions associated with the monounsaturated fatty acids profile in the *Longissimus thoracis* of Nellore

Trait	QTL window	% Variance explained SNP window^a^	PCG^b^
Total MUFA	4:31045067-31056443	1.27	RAPGEF5
	4:10646197-10655217	1.10	CALCR MIR653 MIR489
	15:23027848-23042930	1.77	-^c^
	15:77580057-77608737	1.67	CKAP5
	17:19998159-20045296	1.43	-
C14:1	6:20782674-20793003	1.75	ARHGEF38
	3: 93656044-93678760	2.12	SLC1A7
	3: 106397884-106406683	1.64	COL9A2
C16:1	5: 112777768-112833555	1.73	MIR1281
	5: 114505769-114536452	1.22	TTLL1
	6: 88311624-88350890	1.79	SLC4A4
	12: 1698087-1733249	3.27	TDRD3
	14: 45588151-45625166	1.12	-
	18: 1131250-1147435	1.35	-
	22:22372870-22388066	1.08	-
	28: 34505791-34523077	1.04	-
C18:1 cis-9	3:6449321-6472465	1.29	
	3:60472950-60520103	1.28	
	4:2070739-2116544	1.12	-
	7:85322486-85362698	1.11	XRCC4
	8:101007779-101024030	1.40	PALM2
	12:21265917-21282993	2.15	WDFY2 DHRS12
	23:44473841-44501022	10.59	ADTRP
C18:1 trans-9	4:8337794-8361230	12.00	CDK14
	8:68605036-68625678	1.29	-
	9:78101823-78132552	1.87	-
	12:61362119-61385432	1.12	-
	18:56273509-56313047	1.45	CD37 TEAD2 DKKL1 CCDC155
	24:56810309-56821447	1.36	WDR7
C18:1 trans-11	2: 59116358-59143632	1.78	-
	4: 55957454-55984897	1.23	-
	5: 99332464-99343514	1.06	YBX3
	6: 1976981-2001221	1.30	MARCH1
	10: 39459983-39493584	2.34	-
	10: 88628112-88637230	1.26	ESRRB
	14: 84372868-84383110	1.33	SNTB1
	26: 46130255-46142795	3.16	ADAM12

^a^Window that consists of continuous 10 SNPs

^b^Positional/putative candidate gene

^c^No PCG associated with the trait

**Table 5:** Genomic regions associated with the polyunsaturated fatty acids in the *Longissimus thoracis* muscle of Nellore

Trait	QTL window	% Variance explained SNP window^a^	PCG^b^
C20:4 n-6	3:3469461-3492275	2.15	-^c^
	5:4417952-4432872	3.06	-
	9:84138533-84184280	4.95	FBXO30
	15:27938668-27964533	1.17	SIK3
	16:61647491-61661042	2.34	RALGPS2 ANGPTL1
	19:61671067-62341372	5.01	ABCA5 ABCA6 ABCA10 MAP2K6 PRKAR1A
	21:70498936-70511360	1.72	-
	23:25179729-25202537	4.20	ELOVL5
C18:2 cis9 cis12 n6	1: 71431023- 71499402	4.92	SLC51A PCYT1A TCTEX1D2
	8: 4017767- 4040618	2.06	GALNTL6
	11:82837386- 82851713	3.09	DDX1
	12: 1850108- 1876467	3.38	-
	21:17398974- 17427182	4.04	-
	23:44473841- 44501022	9.33	ADTRP
C18:3 n3	4:111759995- 111777571	2.73	CNTNAP2
	12: 1721955-1759560	2.84	TDRD3
	12: 1848851-1867387	11.49	-
C22:6 n-3	1:35178519-35188850	1.20	-
	3:24528861-24552342	1.46	-
	7:92496213-92524656	1.20	GPR98
	8:108906430-108932062	5.10	-
	12: 1848851-1867387	3.56	-
	23:44473841-44501022	1.63	ADTRP
	24:11304045-11346437	1.49	-
	25:12681505-12706195	1.12	-
	26:45184835-45225853	4.12	-
C20:3 n-6 cis-8 cis-11 cis-14	5: 5499399-5512486	1.31	-
	8: 1640176-1659771	1.72	-
	10:97347302-97359563	1.23	-
	11:62497171-62519808	2.96	PELI1
	15: 7810022-7842535	1.14	-
	20: 1657499-1668052	1.39	FAM196B DOCK2
	25:12681505-12706195	1.80	-
Total PUFA	14:21344791- 21376691	1.33	-
	2:63074224-63103605	2.01	TMEM163
	10:97347302- 97359563	1.52	-
	12:36843604-36886833	6.24	RNF17
	14:80616824- 80631460	1.97	RALYL
	21:17398974-17427182	1.23	AGBL1
	23:44473841-44501022	6.79	ADTRP

^a^Window that consists of continuous 10 SNPs

^b^Positional/putative candidate gene

^c^No PCG associated with the trait

**Table 6:** Genomic regions associated with the omega-3 and omega-6 fatty acids, and the omega-6/omega-3 ratio in the *Longissimus thoracis* muscle of Nellore

Trait	QTL window	% Variance explained SNP window^a^	PCG^b^
Total n-3	3:7439426-7455843	1.14	NOS1AP
	7:92487944-92521811	1.45	GPR98
	8:88219918-88234648	9.99	-^c^
	12:1850108-1876467	3.53	-
	21:17398974-17427182	1.13	LOC100300175
	25:12775670-12817779	1.22	-
Total n-6	2:63074224-63103605	1.68	TMEM163
	3:49887762-49898290	1.41	BCAR3
	10:97347302-97359563	1.69	-
	12:36843604-36886833	5.61	RNF17
	14:80616824-80631460	1.86	RALYL
	23:44473841-44501022	6.68	ADTRP
Ratio of n-6:n-3	1:60602819-60636700	1.11	-
	2:131895441-131915476	1.69	-
	3:53399171-53439878	1.39	ZNF326
	6:28611816-28631205	1.92	-
	10:52660431-52708192	1.01	POLR2M
	10:88145431-88173762	1.01	TTLL5
	16:46586463-46598618	1.78	-
	16:63634797-63657084	1.61	MR1
	29:10816550-10828285	1.16	LOC505383

^a^Window that consists of continuous 10 SNPs

^b^Positional/putative candidate gene

^c^No PCG associated with the trait

In GWAS studies of intramuscular fat and fat deposition in meat of Nellore cattle, using the same marker density as in this study, the authors found 33genomic regions (windows with 1 Mb SNPs) associated with the traits, deposition of intramuscular fat and meat fatty acid profile [13]. Similarly, other GWAS for Angus cattle, using a 54 K genotyping panel, found fifty-seven genomic regions associated with the fatty acids profile trait in meat [16]. For any fatty acid, non-overlapping regions were found with the results obtained by [13, 14, 16], who also performed GWAS for beef FA profile in several breeds. Even though no overlapping regions were found for the same fatty acids, regions in the same chromosome were found, but at the longer distances (>1 Mb). This inconsistent between our results and previous studies could be due the differences between studied populations (breed and environment), distribution of linkage disequilibrium among the causal mutations and genetic markers, model applied to perform the GWAS, genetic marker density. In addition, physiological and metabolic factors involved in those populations might help to explain the differences observed by researchers. In this sense, it is important to highlight that the expression of FA profile in beef is probably influenced by many loci of small effect. Thus, it is expected that each PCG contribute differently for the additive genetic variance of each FA in different populations, environments and management conditions.

### Saturated fatty acids

A total of 31 genomic regions that explain more than 1 % of genotypic were found for total saturated fatty acids, C12:0, C14:0, C16:0 and C18:0. These regions are distributed on chromosomes BTA1, BTA2, BTA3, BTA4, BTA5, BTA7, BTA8, BTA9, BTA12, BTA16, BTA17, BTA20, BTA24, and BTA29 (Table 3 and Additional file 1).

**Fig. 1:**
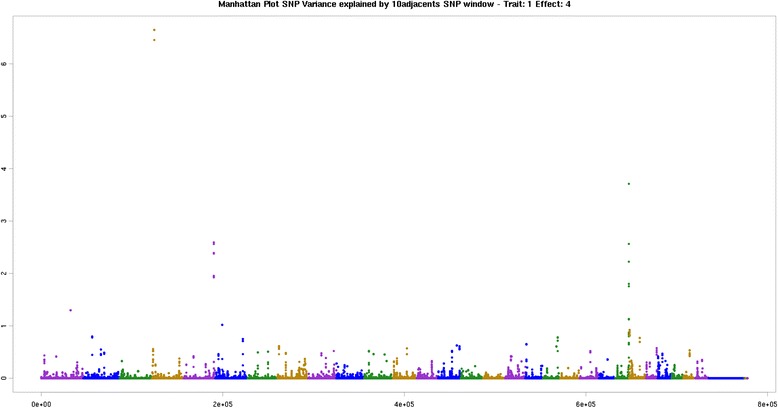
Manhattan plot of the genome-wide association study for sum of SFA in Nellore. The X-axis represents the chromosomes, and the Y-axis shows the proportion of genetic variance explained by windows of 10 adjacent SNPs for total saturated fatty acids in Nellore

The regions identified on BTA1 and BTA5 chromosomes associated with the total saturated fatty acids had no PCG. BTA4 at 83 Mb had a window that explained the highest percentage of additive genetic variance for the saturated fatty acids group (Fig. 1) and an associated candidate gene. The gene in this region is the *CDK14* gene of the cyclin-dependent kinase family (*CDK*). This gene is associated with production of kinase protein, enzymes that catalyze the phosphorylation of proteins by transferring one phosphoryl group of ATP and in exceptional cases, from GTP to threonine, serine (Ser/Thr specific kinase) or tyrosine residues (Tyr specific kinase) [39]. The region found on chromosome BTA23 (Fig. 1) houses the *GMDS* gene and acts on the metabolism of amino sugars and nucleotide sugars.

Three regions on different chromosomes were associated with lauric (C12:0) acid. The BTA7 at 10 MB and BTA29 at 35 MB with no associated candidate genes and BTA16 at 20 MB associated with the ESRRG gene. Studies show that this gene is strongly correlated with the assembly and regulation of other adipogenic genes, and metabolism and transport of lipids [40, 41].

A total of 11 areas in seven different chromosomes (BTA1, BTA2, BTA8, BTA9, BTA12, BTA17, and BTA29) were associated with the myristic (C14:0) acid. Two different regions have been identified for this acid. The first, BTA1 at 71 Mb has three associated genes: *SLC51A*, *PCYT1A*, and *TCTEX1D2*. The main candidate is *PCYT1A*, which is a protein-coding gene controlling the phosphatidylcholine, a phospholipid emulsifier with detergent action that reduces surface tension, forming smaller fat particles as triglycerides [42] and has been related to fat percentage and body mass index in humans [43].

The second region was identified on BTA1 at 112 Mb and associated with the *PLCH1* gene. This gene is a protein-coding, member of the PLC-eta family of the phosphoinositide-specific phospholipase C (*PLC*) superfamily of enzymes that cleave phosphatidylinositol 4,5-bisphosphate (PtdIns(4,5)P2) to generate second messengers inositol 1,4,5-trisphosphate (IP3) and diacylglycerol (DAG). Phospholipases are a group of enzymes that hydrolyze phospholipids into fatty acids and other lipophilic molecules. The PLC enzyme is subdivided into beta, gamma, delta, epsilon, zeta, and eta subtypes, which catalyze the hydrolysis of phosphatidylinositol 4,5-bisphosphate (PIP2) into inositol 1,4,5-trisphosphate (IP3) and 1.2-diacilglicerol (DAG). Phospholipases are usually expressed and have diverse biological functions, including a role in inflammation [44]. On BTA2 at 95 Mb, a candidate gene was associated with the fatty acid C14:0, *ADAM23*, which, among other functions, was found to suppress adipogenesis in mice [45]. BTA8 at 4 Mb had a candidate gene (*GALNTL6*) associated with C14:0. This gene catalyzes the initial reaction in the biosynthesis of oligosaccharides, transferring an N-acetyl-D-galactosamine residue to a serine or threonine residue in the protein receptor [46]. The *PEX7* gene was identified on BTA9 at 75 Mb. This gene encodes cytosolic receptor for the set of enzymes of peroxisomal matrix targeted to the organelle by the PTS2. Mutations in this gene cause disorders in peroxisome biogenesis, which are characterized by multiple modifications in the peroxisome function [47].

Eight regions on different chromosomes were identified for the palmitic (C16:0) acid, BTA1 at 71 Mb, BTA2 at 12 Mb, BTA3 at 6 Mb, BTA4 at 27 Mb, BTA8 at 4 Mb, BTA9 at 7 Mb, BTA12 at 21 Mb and BTA20 at 1 Mb. The first region found (BTA1 at 71 Mb) was exactly the same QTL region found for the saturated fatty acid C14:0 and associated with the same genes (*SLC51A*, *PCYT1A*, and *TCTEX1D2*). The larger genomic region found for the group of saturated fatty acids is on BTA3 at 6 Mb (Table 3). This region had only 1.63 % of the additive genetic variance for the trait C16:0, in which three candidate genes were located: *UAP1*, *UHMK1*, and *HSD17B7*. The *HSD17B7* encodes an enzyme with the same function of the 17-beta-hydroxysteroid dehydrogenase (EC 1.1.1.62) in sex steroid biosynthesis and 3-ketosteroid reductase (EC 1.1.1.270), and acts on the biosynthesis of cholesterol [48]. This gene is expressed in most lineages of fast-growing broilers [49] and is found in the subcutaneous and omental adipose tissue in humans [50].

The region identified on BTA8 at 4 Mb for myristic (C14:0) acid, where the same *GALNTL6* gene described above was located. The *BAI3* gene identified on BTA9 at 7 Mb is known to participate in the myoblast fusion; it is present in the extracellular matrix and plays a role in the adipose tissue formation [51]. The BTA12 at 21 Mb QTL was associated with two PCGs: *ATP7B* and *DHRS12*. The *ATP7B* gene participates in the regulation of copper in the body. Mice with this non-functional gene present altered cholesterol and fatty acids synthesis [52]. The *DHRS12* encodes a member of the dehydrogenase/reductase short-chain family. Members of this family are enzymes that metabolize many compounds, such as steroid hormones, prostaglandins, retinoids, lipids and xenobiotics [53] and a deletion of the region where the gene is associated with lipomas in humans [54]. BTA20 at 1 Mb was associated with two PCG: *FAM196B* and *DOCK2*, the latter is involved in actin cytoskeleton remodeling through activation of RAC GTPase [55] and interacts with various lipids [56].

Five different regions were associated with stearic (C18:0) acid and only one, BTA3 at 115 Mb, was not identified with any PCG. The candidate gene *FNDC3B* was located on BTA1 at 96 Mb while the *EPB41* gene, on BTA2 at 125 Mb. Two PCGs were identified on BTA 8 at 100 Mb: transmembrane protein 245 (*TEMEM245*) and microRNA 32 (*MIR32*). However, none of these genes has a described function in lipid metabolism.

### Monounsaturated fatty acids (MUFA)

A total of 37 genomic regions, distributed over sixteen different chromosomes, account for more than 1 % of the genetic variance for monounsaturated fatty acids, which relates to total monounsaturated fatty acids, C14:1, C16:1, C18:1 trans11, C18:1 cis9 and C18:1 trans9 (Table 4 and Additional file 1).

In the first region of BTA4 at 31 Mb, the *RAPGEF5* gene was associated with total monounsaturated fatty acids. In the second region, all three genes were identified: *CALCR*, *MIR653*, and *MIR489*. The *CALCR* gene expression is associated with the production of various lipids in humans [57] and has SNPs associated with the production of milk fat and body condition in dairy cattle [58]. The BTA15 at 77 Mb was associated with the *CKAP5* gene. No PCG was identified on BTA17 at 19 Mb, and BTA15 at 23 Mb. The C16:1 FA was associated to ten different regions where six PCG were found in. For the first region, BTA3 at 93 Mb, the gene *SLC1A* was identified. The family of this gene includes five high-affinity glutamate transporters, *EAAC1*, *GLT*-*1*, *GLAST*, *EAAT4* and *EAAT5* (*SLC1A1*, *SLC1A2*, *SLC1A3*, *SLC1A6*, and *SLC1A7*, respectively), also known as excitatory amino acid transporters (EAATs), which are sodium and potassium-dependent members of the solute carrier family 6 (*SLC1*) found widely distributed in the whole brain [59]. On BTA3 at 106 Mb one PCG was identified, *COL9A2*, which is related to extracellular matrix structural constituent conferring tensile strength according to gene ontology (GO:0030020).

**Fig. 2:**
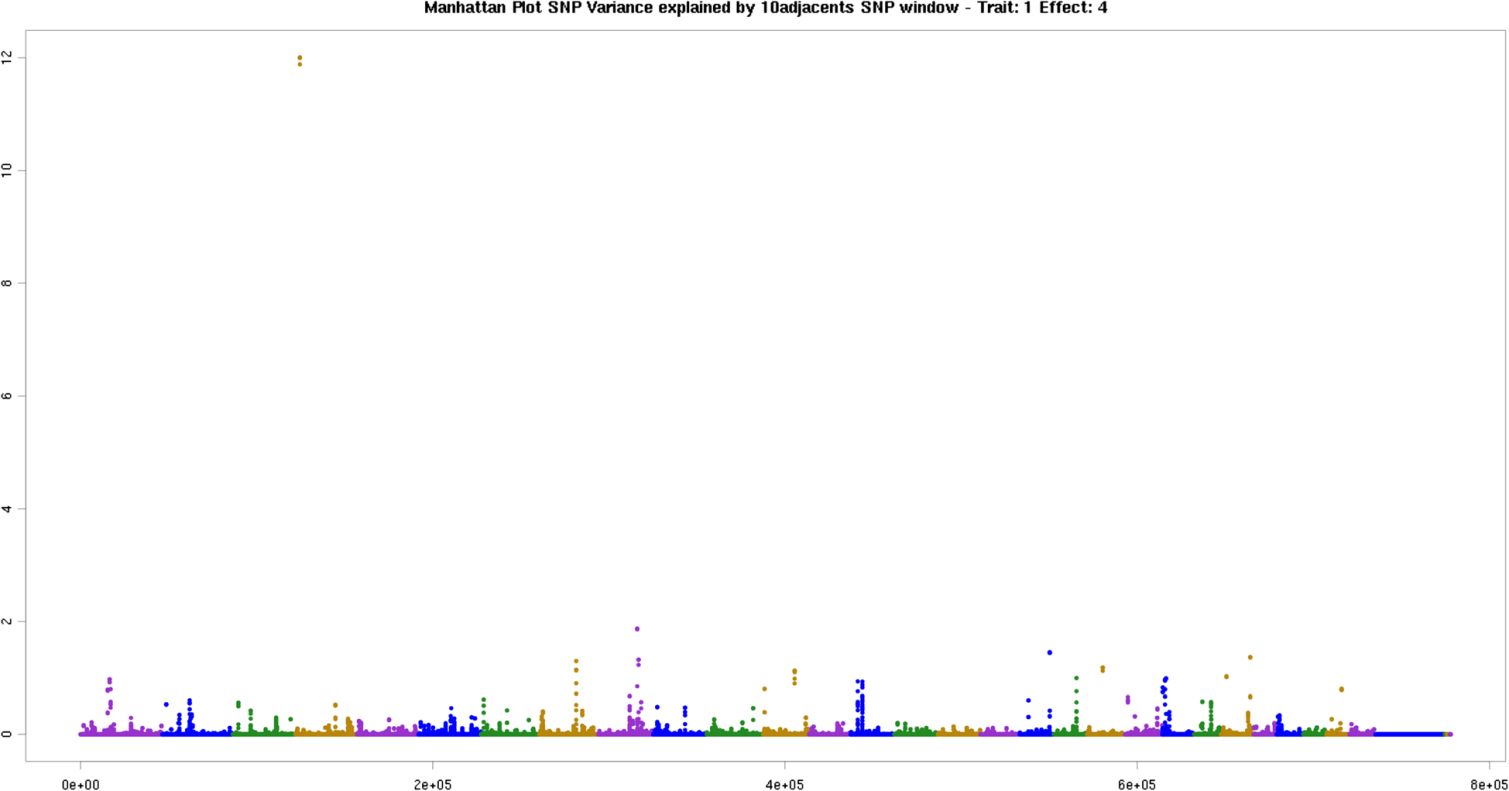
Manhattan plot of the genome-wide association study for C18:1n9t (elaidic) in Nellore. The X-axis represents the chromosomes, and the Y-axis shows the proportion of genetic variance explained by windows of 10 adjacent SNPs elaidic fatty acids in Nellore

On BTA3, at 6 Mb and 60 Mb, BTA4 at 2 Mb, BTA7 at 85 Mb and BTA8 at 101 Mb, BTA12 at 21 Mb and BTA23 at 44 MB were associated with oleic acid (C18:1 cis 9). BTA7 at 85 Mb was associated with the PCG, *XRCC4*. Studies shows that this gene functionally complements XR-1 Chinese hamster ovary cell mutant, which is impaired in DNA double-strand breaks produced by ionizing radiation and restriction enzymes [60]. On BTA8 at 101 Mb, the PCG *PALM2* was identified. Two genes were identified on BTA12 at 21 Mb: *WDFY2* and *DHRS12*. On BTA23 at 44 Mb, the *ADTRP*. The genes *DHRS12* and ADTRP were the same reported for C16:0, thus are described above. The genomic region that explained most of the genetic variance of the unsaturated fatty acids group was located on BTA4 at 8 Mb, where the *CDK14* gene was associated with the C18:1 trans-9 (elaidic acid) (Fig. 2). The same region was found for total saturated fatty acids (Table 3), where the *CKD14* gene is associated with the same two traits described above. On BTA18 at 56 Mb, four genes associated with the above traits were identified: *CD37*, *TEAD2*, *DKKL1* and *CCDC155*.

The vacenic fatty acid (C18:1 trans 11) was associated with eight regions in seven different chromosomes. One of these regions, on BTA10 at 88 Mb, was the same associated with lauric (C12:0), thus the PCG *ERSSB* gene was described above.

### Polyunsaturated fatty acids (PUFA)

Forty genomic regions explained more than 1 % of the additive genetic variation for the polyunsaturated fatty acids group, as: C20:4 n-6, C18:2 cis-9 cis12 n-6, C18:3 n-3, C22:6 n-3 and C20:3 n-6 cis-8 cis-11 cis-14 (Table 5 and Additional file 1).

On BTA2 at 63 Mb, the *TMEM163* gene was associated with the total PUFA. *TMEM* 163 variants were associated with decreased fasting plasma insulin and also with the homeostatic model assessment of insulin resistance, indicating plausible effect through impaired insulin secretion [61]. The fatty acid C20:4 n-6 was associated with eight different genomic regions (Table 5). On BTA9 at 84 Mb, the *FBXO30* gene was associated with arachidonic acid. BTA19 at 61 Mb (the largest region found for the group of polyunsaturated fatty acids) had five associated genes: *ABCA5*, *ABCA6*, *ABCA10*, *MAP2K6*, and *PRKAR1A*. The genes of the ABC Group are associated with cholesterol metabolism and lipids homeostasis [62], as well as *ABCA5*, *ABCA6* and *ABCA10* [63, 64]. The *MAP2K6* gene was associated with backfat thickness, marbling score and carcass weight in Hanwoo cattle [65] and identified in two selection signatures in Galloway and Gelbvieh cattle [66]. The *PRKAR1A* is involved in the regulation of lipid and glucose metabolism and is a component of the signal transduction mechanism of certain GPCRs (G-protein coupled receptor) [67]. Mutations in this gene have been associated with obesity phenotypes in humans [68].

On BTA23 at 25 Mb, the *ELOVL* fatty acid elongase 5 (*ELOVL5*) gene was associated with the fatty acid C20:4 n-6. This gene plays an important role in the elongation of saturated and monounsaturated fatty acids up to 24 carbons (GO:0009922), condensing enzymes that catalyze the synthesis of monounsaturated and polyunsaturated fatty acids of very long chains, specifically the current polyunsaturated acyl-CoA with higher activity C18:3(n-6) acyl-CoA [69]. In mammals, seven enzymes have been identified in the *ELOVL* family (*ELOVL1*-*7*). Each *ELOVL* enzyme has a distinct distribution in different tissues, and different enzymes exhibit different preferences for the fatty acid substrate. The *ELOVL5* and *ELOVL6* genes are involved in the production/synthesis of palmitic (C16:0), palmitoleic (C16:1), stearic (C18:0) and oleic (C18:1) fatty acids, important beef fatty acids. Therefore, the role of *ELOVL5* and *ELOVL6* genes in the synthesis of these fatty acids is of great importance in beef breeding programs [70, 71].

**Fig. 3:**
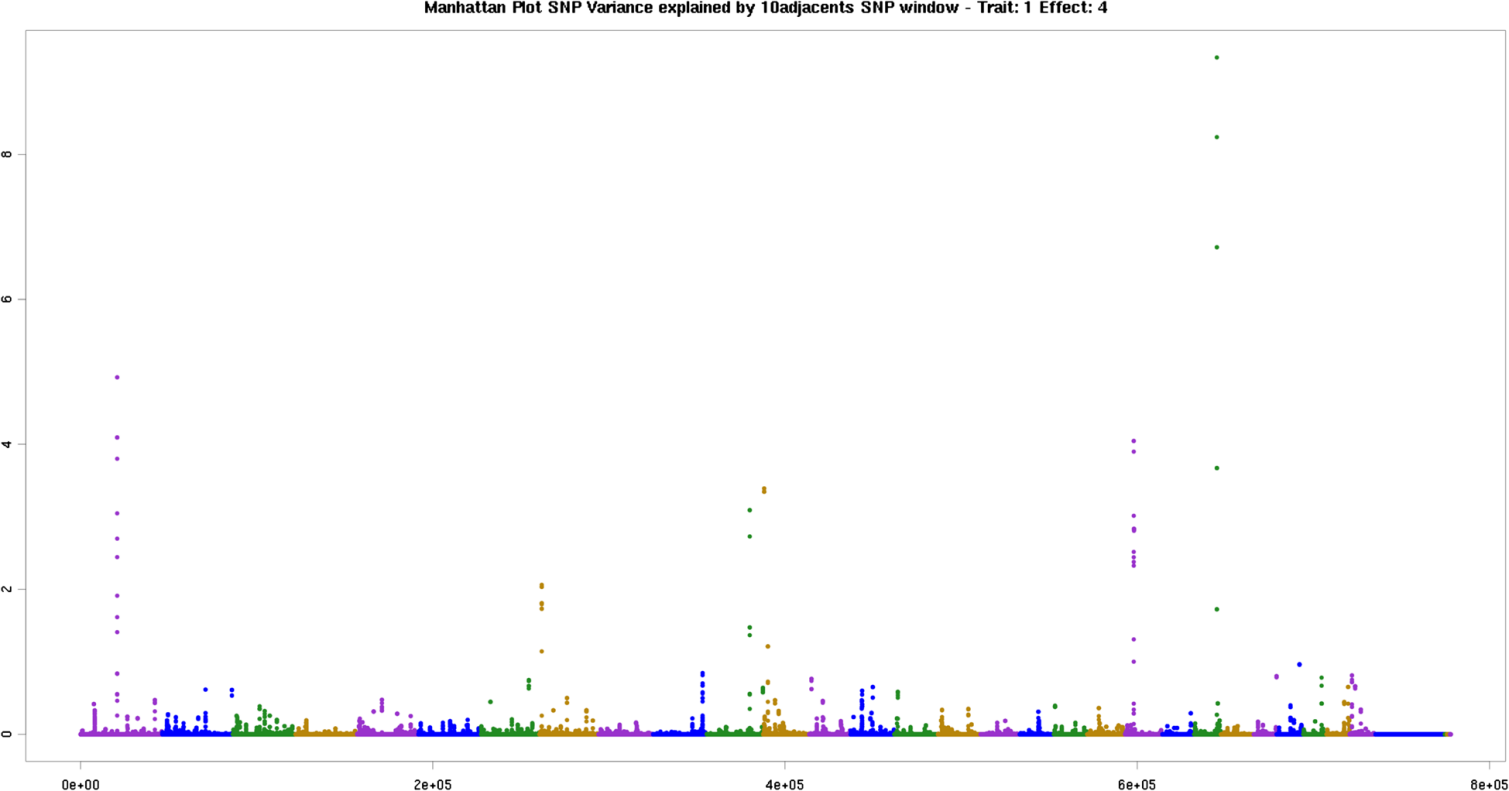
Manhattan plot of the genome-wide association study for C18:2n6 (linoleic) in Nellore. The X-axis represents the chromosomes, and the Y-axis shows the proportion of genetic variance explained by windows of 10 adjacent SNPs linoleic fatty acids in Nellore

The genomic regions of BTA1 at 71 Mb, BTA8 at 4 Mb, BTA11 at 82 Mb, BTA12 at 1 Mb, BTA21 at 17 BM and BTA23 at 44 Mb were associated with the linoleic (C18:2 cis-9 cis-12; n-6) acid. The two regions, BTA1 at 71 Mb and BTA8 at 4 Mb were similar to those observed for the saturated fatty acids C14:0 and C16:0, where the *SLC51*A, *PCYT1A*, *TCTEX1D*, and *GALNTL6* genes have been previously described. The BTA23 at 44 Mb position had a candidate gene associated and explaining a important proportion of the additive genetic variance of the polyunsaturated fatty acids group (Fig. 3). This same region had the *ADTRP* gene associated with total polyunsaturated fatty acids. The BTA11 at 82 MB was associated with C18:2 cis-9 cis-12 n-6, (gene: *Asp*-*Glu*-*Ala* -*Asp*) box helicase 1 (DDX1), which acts as a RNA helicase dependent of ATP, able to relax both RNA-RNA and DNA-RNA duplexes [72].

BTA4 at 111 Mb and BTA12 at 1 Mb were associated with the linolenic (C18:3 n-3.) BTA4 at 111 Mb was associated with the *CNTNAP2* gene, which implies diet-induced obesity [73]. The gene tudor domain containing 3 (*TDRD3*) was identified on BTA12 at 1 Mb [74].

Nine QTL regions were associated with C22:6 n-3 FA, but PCGs were found in only two of these regions: BTA7 at 92 Mb and BTA23 at 44 Mb. BTA7 at 92 Mb was associated with the *GPR98* gene. This gene encodes a member of the receptors superfamily coupled to the G protein. This encoded protein contains a seven-transmembrane receptor domain bound to calcium. It is expressed in the central nervous system [75] and associated with the body condition score in humans [60]. BTA23 at 44 Mb was also associated with the fatty acid C18:2 cis-9 cis-12 n-6 and, therefore, PCG *ADTRP* has been described above.

The fatty acid C20:3 n-6 cis-8 cis-11 cis-14 was associated with seven regions in seven different chromosomes, but PCGs were identified in only two regions: BTA11 at 62 MB and BTA20 at 1 Mb. The PCG *Pellino E3* ubiquitin protein ligase 1 (*PELI1*) was identified on BTA11 at 62 Mb. BTA20 at 1 Mb had two PCGs identified: family with sequence similarity 196, member B (*FAM196BI*) and dedicator of cytokinesis 2 (*DOCK2*). *DOCK2* is involved in cytoskeletal rearrangements necessary for lymphocyte migration in response to chemokines. This PGC activates *RAC1* and *RAC2*, but not the *CDC42*, because it acts as a guanine exchange factor (GEF) nucleotide that changes the *GDP* to free GTP [55].

### Omega 3 and 6 fatty acids

A total 21 genomic regions accounted for more than 1 % of the genetic variance for n-3 and n-6 fatty acids, and the n-6:n-3 ratio (Table 6 and Additional file 1).

On BTA3 at 7 Mb, BTA7 at 92 Mb, BTA8 at 88 Mb, BTA12 at 1 Mb, BTA21 at 17 Mb, and BTA25 at 12 Mb were associated with total n-3 fatty acids. On the other hand, no PCGs were identified on BTA8 at 88 Mb, BTA12 at 1 Mb, and BTA25 at 12 Mb. Moreover, BTA3 at Mb 7 Mb and BTA7 at 92 were also associated with the C18:1 cis-9 and C22:6 n-3 fatty acids (FA) and, therefore, with the *NOS1AP* and *GPR98* genes described above, respectively.

**Fig. 4:**
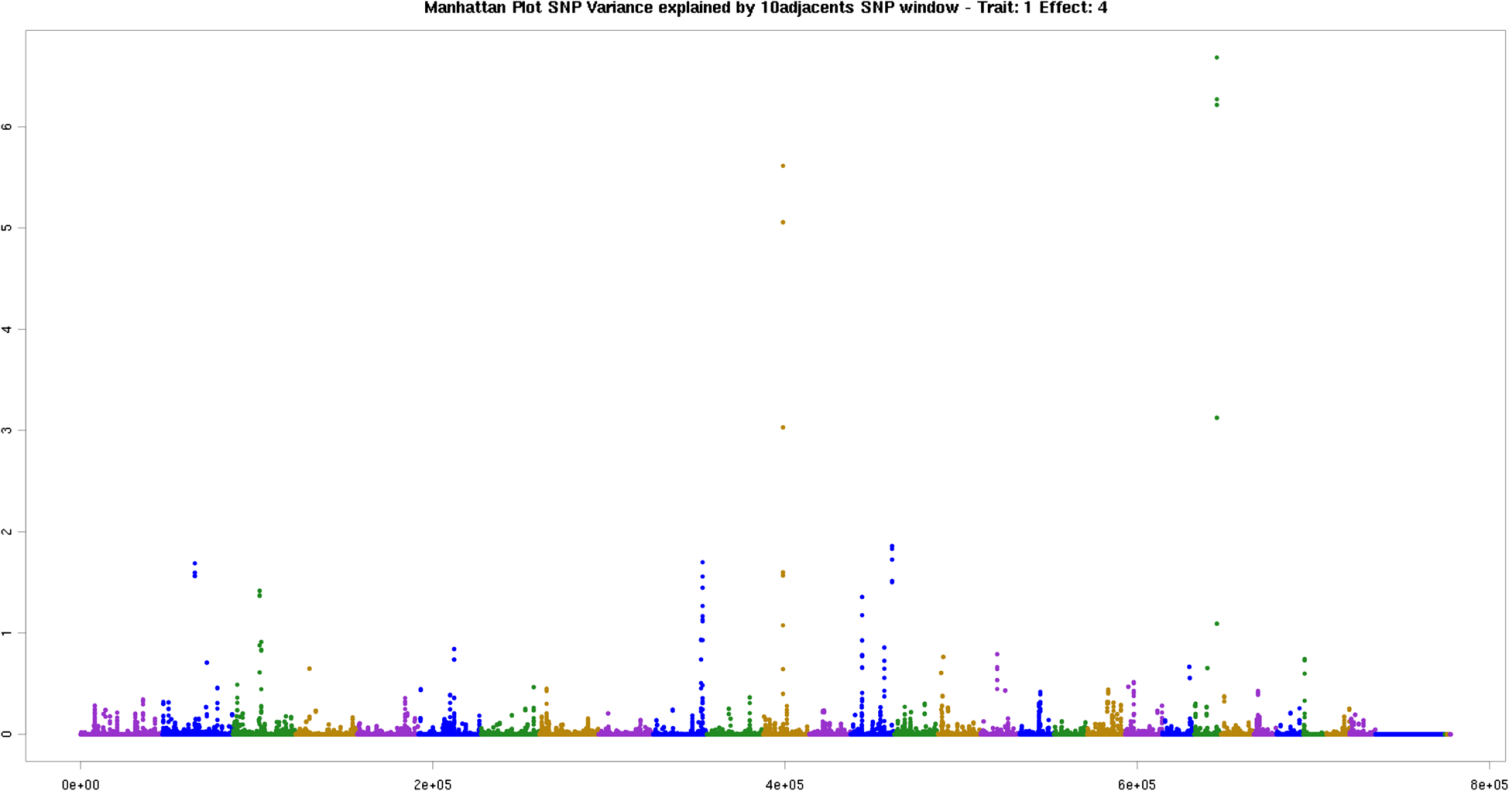
Manhattan plot of the genome-wide association study for sum omega-6 in Nellore. The X-axis represents the chromosomes, and the Y-axis shows the proportion of genetic variance explained by windows of 10 adjacent SNPs omega-6 fatty acids in Nellore

On BTA2 at 63 Mb, BTA3 at 49 Mb, BTA10 at 97 Mb, BTA12 at 36 Mb, BTA14 at 80 Mb and BTA23 at 44 Mb were associated with total n-6 fatty acids. The BTA2 at 63 Mb harbors the gene transmembrane protein 163 (*TMEM163*). This same region has also been associated with total polyunsaturated fatty acids (PUFA). The BTA3 at 49 Mb was the largest region associated with the omega fatty acids group, where the *BCAR3* gene is found. This gene has been linked to breast cancer in humans [76]. BTA12 at 36 Mb, BTA14 at 80 Mb and BTA23 at 44 Mb have been associated previously with other long-chain fatty acids, whereas the latter region explained the greatest proportion of variance (Fig. 4). BTA12 at 36 Mb and BTA14 at 80 Mb were also associated with total polyunsaturated fatty acids and the genes already described. BTA23 at 44 Mb was also associated with the polyunsaturated FAs: C18:2 cis-9 cis-12 n-6, C22:6 n-3 and total PUFA.

The single step method method allows to combine the information of genotyped and non-genotyped animals in the genetic evaluation process, thus expanding the scope and identification of potential regions associated with loci responsible for variations in the studied traits [77]. In this sense, some authors [78] compared different GWAS methodologies and reported that the single step method method partitions the genetic variance for a particular region among all SNPs markers. On the other hand BayesB or BayesCpi methods, for example, penalizes the zero regions and tends to overestimate the genetic variability explained for each of the identified regions. There is some controversy regarding the capacity or ability of different methods to identify genomic regions associated with the phenotype [20–22]. Therefore, caution should be applied when interpreting results from different populations, using various methods because the identified associations depend on the strength or magnitude of the association, methodology, and implementation details.

In this study, we found several nearby areas of major QTL associated with groups of saturated, monounsaturated, and polyunsaturated fatty acids, in Nellore meat. These regions harbor interesting PCG, which are involved in lipid metabolism, as a constituent of cell membranes, receptors for reproductive hormones, biosynthesis and hydrolysis of phospholipids and membrane constituents, synthesis of protein kinases, transport and use of fatty acids and cholesterol, energy metabolism, elongation factors and synthesis of long-chain fatty acids in different species. Among the many genes identified, the *ELOVL5* gene located on chromosome BTA23 at 25 Mb and associated with the C20:4 n-6 (arachidonic acid) is highlighted. The genes responsible for the elongation of very-long-chain fatty acid (*ELOVL*) encode enzymes that play an important role in the elongation of long-chain fatty acids. The fatty acid synthesis involves a number of enzymes, such as fatty acid synthase (*FASN*), which is located on chromosome BTA19 between 51.384.922 and 51.403.614 bp, while its variations have been related to fatty acid composition of Angus beef [79]. In mammals, *FASN* synthesizes the fatty acids that contain up to 16 carbon atoms, and the genes of the *ELOVLs* group produce long-chain fatty acids with 18 carbon atoms or more [80, 81].

The results of the present study pointed out some PCG that were found associated to several FA of different saturation state: the *CDK14* gene was associated with C18:1 trans-9 and SFA; the *TMEM163* gene related to n-6 fatty acids and PUFA; and the *SLC51*A, *PCYT1A*, *TCTEX1D*, and *GALNTL6* genes that influence the linoleic acid and also some individual saturated fatty acids, like the C14:0 and C16:0. These results could be due to pleitropic effects, where the expression of differents FA could influenced by the same gene which acts in a coordinate manner to contribute to the synthesis of beef FA. Similar findings were reported by [16], where both genes, *FASN* and *THRSP*, exhibited pleiotropic effect for the most studided FA in Angus cattle. Genes involved in muscle lipid composition of 15 european Bos taurus breeds were studied by [82], which reported pleiotropic effects for genes like *CRI1*, *DGAT1*, *FOXO1*, *MMP1*, *SOC2* and *NEB*, affecting several beef FA.

The large number of genomic regions associated with the fatty acid profile found in this study should help to understand the genetic and metabolic mechanisms that determine the fatty acids profile of intramuscular fat, especially in zebu. According to the results of this study, the meat fatty acid profile of Zebu is probably controlled by several QTL of small effect and, therefore, the identification of relevant genes or large effect seems to be difficult, since for most fatty acids, the contribution of each region or window for the additive genetic variation was small. Therefore, strategies such as genomic selection using or considering the variability among markers at the same time would be more appropriate to improve the fatty acid profile of the bovine meat. The database used in the study is broad since it contains animals that participate in beef cattle breeding programs, and breeders that are sold and used in various regions of the country. Therefore, the results should contribute to the selection and improvement of the meat quality from zebu raised in tropical conditions.

## Conclusion

Several genomic regions associated with QTL related to lipid metabolism and fatty acid composition were identified. The identification of such regions and the respective candidate genes associated with lipid metabolism and energy transport hormones such as, for example, *ELOVL5*, *ESSRG*, *PCYT1A* and genes of the *ABC* group (*ABC5*, *ABC6* and *ABC10*), should contribute to improve the genetic knowledge regarding the fatty acids profile of Nellore (*Bos indicus*) and help to improve the selection of such traits to favor human health. In addition, these regions can be used in future fine mapping studies, whose primary function is to search for informative causative mutations. These polymorphisms can be inserted into customized low-density chips that assist a more cost-effective genetic evaluation.

## Methods

### Local, animals and management

This study was approved by ethics committee of the Faculty of Agrarian Sciences and Veterinary, Sao Paulo State University (UNESP).

The database contains animal data from eight farms located in the Southeast, Northeast and Midwest of Brazil, which are part of breeding cattle programs. Genotypes (*n* = 1616) and phenotypes (*n* = 963) of Nellore bulls, with average initial age of 24 months, were used. The animals belonged to eight different farms located in the Southeast, Northeast and Midwest regions of Brazil, which participate in three beef cattle breeding programs (NeloreQualitas, Paint and DeltaGen). In these breeding programs animals are selected based on growth, finishing and sexual precocity traits.

Breeding seasons are adopted at different periods on these farms. Therefore calving seasons concentrate from August to October in some farms and from November to January in others, and weaning was performed at seven months of age. The animals were raised on grazing conditions using *Brachiaria sp*. and *Panicum sp* forages, and free access to mineral salt, at density varying from 1.2 to 1.6 animal unit/hectare (AU/ha. After yearling, the breeding animals were selected and the rest remained in feedlot. During feedlot, the forage: concentrate ratio ranged from 50:50 to 70:30, depending on the farm. In general, whole-plant corn or sorghum silage was used as high quality forage. Grains of corn and/or sorghum, and soybeans, soybean meal, or sunflower seeds were used as protein concentrate. The criteria used by farmers for slaughtering was weight (500–550 kg). After stored for 48 h at 0-2 °C, meat samples were removed from the *Longissimus thoracis* muscle, between the 12 and 13th ribs from each animal, placed in plastic bags and stored at −80 °C until further analysis to determine the fatty acid profile. The percentage of lipids in the *Longissimus thoracis* muscle (IMF) was obtained using the method proposed by [83].

### Determination of the fatty acid profile

The total lipid concentration was quantified at the Animal Product Technology Laboratory in the Technology Department of FCAV/Unesp using the Bligh and Dyer [84] method. The meat fatty acids were extracted using the method of Folch et al. [83] and the methyl esters were formed according to Kramer et al. [85]. The fatty acid profile was determined at the Meat Science Laboratory (LCC) in the Department of Animal Nutrition and Production at FMVZ/USP, using the extraction method by Folch et al. [83]. Muscle samples (~100 g) were collected and ground to determine the fatty acid profile. The lipids were extracted by homogenizing the sample with a chloroform and methanol (2:1) solution. NaCl at 1.5 % was added to isolate the lipids.

The separated fat was methylated, and the methyl esters were formed according to Kramer et al. [85]. The fatty acids were quantified by gas chromatography (GC-2010 Plus - Shimadzu AOC 20i auto-injector) with a 100 m SP-2560 capillary column (0.25 mm in diameter with 0.02 mm thickness, Supelco, Bellefonte, PA). The initiating temperature of 70 °C was increased gradually up to 175 °C (13 °C/min), held for 27 min, and increased further up to 215 °C (4 °C/min) and held for 31 min. Hydrogen (H2) was the carrier gas, with 40 cm3/s. Fatty acids were identified by comparing the retention time of methyl esters of the samples with the standards C4-C24 (F.A.M.E mix Sigma®), vaccenic acid C18:1 trans-11 (V038-1G, Sigma®) C18:2 trans-10 cis-12 (UC-61 M 100 mg), CLA e C18:2 cis-9, trans-11 (UC- 60 M 100 mg), (Sigma®) and tricosanoic acid (Sigma®). Fatty acids were quantified by normalizing the area under the curve of methyl esters using the GS solution 2.42 software. Fatty acids were expressed as a percentage of the total fatty acid methyl ester. The fatty acid profile in meat was performed at the Meat Science Laboratory (LCC) in the Department of Animal Nutrition and Production at FMVZ/USP.

The following individual fatty acids were selected: lauric (C12:0), myristic (C14:0), palmitic (C16:0), stearic (C18:0), myristoleic (C14:1), palmitoleic (16:1), oleic (C18:1 *cis*-9), elaidic (C18:1 trans9), CLA-*cis* (C18:2c9t11), CLA-*trans* (C18:2 trans10 cis 12), vaccenic (C18:1 trans11), linoleic (C18:2 cis9Cis12n6), docosahexaenoic (DHA) (C22:6 n3), and eicosatrienoic FA (C20:3 n6 cis-8,11,14). These FA were chosen due to their importance to human health and their high content in animals from confinement, such as the oleic acid. The sum of saturated (C10:0 + C11:0 + C12:0 + C13:0 + C14:0 + C15:0 + C16:0 + C17:0 + C18:0 + C21:0 + C24:0), monounsaturated (C16:1 + C17:1 c10 + C18:1 t11 + C15:1 c10 + C20:1 c11 + C24:1 + C22:1 n9 + C18:1n9c + C14:1 + C18:1 n9t), polyunsaturated (C18:2 n6 + C18:3 n3 + C18:3 n6 + C20:3 n3 cis-11, 14, 17 + C20:3 n6 cis-8, 11, 14 + C20:4 n6 + C20:5 n3 + C22:6 n3), omega 6 (C18:3 n6 + C20:3 n6 c8, c11, c14 + C18:2 n6 + C20:4 n6) and omega 3 (C18:3 n3 + C20:3 n3 c11, c14, c17 + C22:6 n3 + C20:5 n3) were calculated. The polyunsaturated/saturated fatty acids and n-6/n-3 ratios were also calculated.

### Genotyping of animals

A total of 1616 animals were genotyped using 777,962 SNPs of the Bovine SNP BeadChip (High-Density Bovine BeadChip). The quality control of the SNPs markers consisted of excluding those with unknown genomic position, located on sex chromosomes; monomorphic and markers with minor allele frequency (MAF) less than 0.05; call rate less than 90 %, and markers with excess heterozygosity. Samples with a call rate less than 90 % were also excluded. After quality control, 470,007 SNPs from 1,556 animal samples were left.

### Analysis of the genomic association

To perform the GWAS analyses the single-step GBLUP (ssGBLUP) method was applied. The ssGBLUP model is a modification of BLUP with numerator relationship matrix A^−1^ matrix replaced by H^−1^ [86]:

$$ {H}^{-1}={A}^{-1}+\left[\begin{array}{cc}\hfill 0\hfill & \hfill 0\hfill \\ {}\hfill 0\hfill & \hfill \kern2em {G}^{-1}-{A}_{{}^{22}}^{-1}\hfill \end{array}\right] $$

Where, *A*_*22*_ is a numerator relationship matrix for genotyped animals, and *G* is a genomic relationship matrix. The genomic matrix can be created following [87] as:

$$ G=ZDZ\hbox{'}q $$

Where, *Z* is a matrix of gene content adjusted for allele frequencies; *D*, a weight matrix for SNP (initially *D* = I); and q, a weighting/normalizing factor. According to Vitezica et al. [88], this factor can be derived by ensuring that the average diagonal in *G* is close to that of *A*_*22*_. The SNP effects and weights for GWAS were derived as follows [21]:

1. Let D = I in the first step.
2. Calculate
3. Calculate GEBVs for the entire data set using ssGBLUP.
4. Convert GEBVs to SNP effects $\widehat{\mathbf{u}}=\frac{{\boldsymbol{\upsigma}}_{\mathbf{u}}^{\mathbf{2}}}{{\boldsymbol{\upsigma}}_{\mathbf{a}}^{\mathbf{2}}}\mathbf{D}\mathbf{Z}\mathbf{\hbox{'}}\mathbf{G}{*}^{\mathbf{\hbox{-}}\mathbf{1}}{\widehat{\mathbf{a}}}_{\mathbf{g}}=\mathbf{D}\mathbf{Z}\mathbf{\hbox{'}}{\left[\mathbf{Z}\mathbf{D}\mathbf{Z}\mathbf{\hbox{'}}\right]}^{\mathbf{\hbox{-}}\mathbf{1}}{\widehat{\mathbf{a}}}_{\mathbf{g}},$ where ${\widehat{\mathbf{\mathsf{a}}}}_{\mathbf{g}}$ is the GEBV of the animals which were also genotyped.
5. Calculate the weight for each SNP: **d**_**i**_ = **û**_**i**_^**2**^**2p**_**i**_(**1** ‐ **p**_**i**_), where i is the i-th SNP
6. Normalized SNP weight to remain the total genetic variance constant.
7. Loop to 2.

The SNP weights were calculated iteratively looping trough steps 4–6. The iterations increase the weights of SNP with large effects and decrease those with small effects.

The percentage of genetic variance explained by i-th region was calculated by:

$$ \frac{Var\left({a}_i\right)}{\sigma_a^2}=\times 100=\frac{Var\left({\displaystyle {\sum}_{j=1}^{10}{Z}_j{\widehat{u}}_j}\right)}{\sigma_a^2}\times 100 $$

Where, *a*_*i*_ is genetic value of the *i*-th region that consists of continuous 10 adjacent SNPs, *σ*_*a*_^2^, the total genetic variance; *Z*_*j*_, the vector of gene content of the *j*-th SNP for all individuals; and û_j_, marker effect of the *i*-th SNP within the *i*-th region.

### Quantitative genetic analysis

The contemporary groups included animals born on the same farm and year, and from the same management group at yearling. The contemporary groups that contained less than three observations and those that deviated 3 standard deviations from the mean of that group were eliminated. The model used for the variance component estimation included random additive direct genetic effect, the fixed effect of the contemporary groups, and the animal’s slaughter age as a covariable (linear and quadratic effect).

The variances components and genetic parameters were estimated using the Bayesian inference [89], considering a linear animal model (ssGBLUP), and the GIBBS2F90 computer programs [32, 33]. The statistical model can be represented by the following matrix form:

$$ y=X\beta +Za+e, $$

where: *y* is the vector of observations; *β*, the vector of fixed effects; *a*, the vector of direct additive genetic effects; *X*, the known incidence matrix; *Z*, the incidence matrix of the random additive direct genetic effect (associates vector *β* with vector *y*); and *e*, the vector of the residual effect. The *priori* distributions of vectors *y*, *a* and *e* were given by:

$$ \begin{array}{l}y\sim MVN\left(X\beta +Za\right)\hfill \\ {}a\Big|G\sim MVN\left(0,H\otimes G\right)\hfill \\ {}e\Big|R\sim MVN\left(0,I\otimes R\right)\hfill \end{array} $$

Where: *H* is the matrix of kinship coefficients between animals obtained from the single-step analyses; R, the matrix of residual variance; I, the identity matrix; G, matrix of additive genetic variance; and, ⊗, the Kronecke product. The prior distribution of variance components of the genetic and residual effects was an inverted Wishart. Uniform initial distribution was defined for fixed effects.

The analyses generated chain lengths of 1,000,000 interactions, where the first 80,000 interactions were discarded. To estimate the parameters, the samples were stored at every 100 cycles, building samples with 800,000 samples. The data convergence was verified with the graphical evaluation of sampled values versus interactions according to the criteria proposed by several authors [90–92], using the Bayesian Output Analysis (BOA) of the R 2.9.0 software [93].

### Searching for genes

The segments that explained values equal to or greater than 1 % of the additive genetic variance were selected to determine the possible QTL regions. The selected segments were identified and located in the bovine genome by surveying the database available in the “National Center for Biotechnology Information” [94] in UMD3.1 version of the bovine genome and Ensembl Genome Browser [95]. In these databanks, it was possible to identify segments located within or close to genes that could explain the variability in the expression of such traits. The classification of genes regarding their biological function was performed on the website “The Database for Annotation, Visualization and Integrated Discovery (DAVID) v. 6.7” [96]. Whereas the already described QTLs were researched on the AnimalQTLdb website [97].

### Availability of supporting data

The data sets supporting the results of this article are included within the article and its additional files.

## Additional file

Additional file 1: Manhattan plot of the genome-wide association study for fatty acids in Nellore. The X-axis represents the chromosomes, and the Y-axis shows the proportion of genetic variance explained by windows of 10 adjacent SNPs in the following 18 fatty acids: arachidonic, CLA-cis, CLA-trans, docosahexaenoic, eicosatrienoic,myristoleic, MUFA, PUFA, myristic, n6:n3, oleic, omega-3, palmitic, stearic, palmitoleic and PUFA:SFA ratio in Nellore. (ZIP 1395 kb)

## Abbreviations

BTA: *Bos Taurus* chromosome.
CLA: Conjugated linoleic acid
FA: Fatty acid
GEBVs: Genomic estimated breeding values
GO: Gene ontology
GWAS: Genome-wide association study
MAF: minor allele frequency
MUFA: sum of monounsaturated fatty acids
n-3: Sum of omega 3 acids
n-6: Sum of omega 6 acids
PCG: Putative candidate gene
PUFA: Sum of polyunsaturated fatty acids
QTL: Quantitative trait loci
SFA: Sum of saturated fatty acids
SNP: Single nucleotide polymorphism
UK: United Kingdom
