# Supplementary figures and images for: Genome-wide association between single nucleotide polymorphisms with beef fatty acid profile in Nellore cattle using the single step procedure
Source: BMC Genomics. 2016 Mar 9;17:213. doi: 10.1186/s12864-016-2511-y (PMC4784275; doi:10.1186/s12864-016-2511-y)

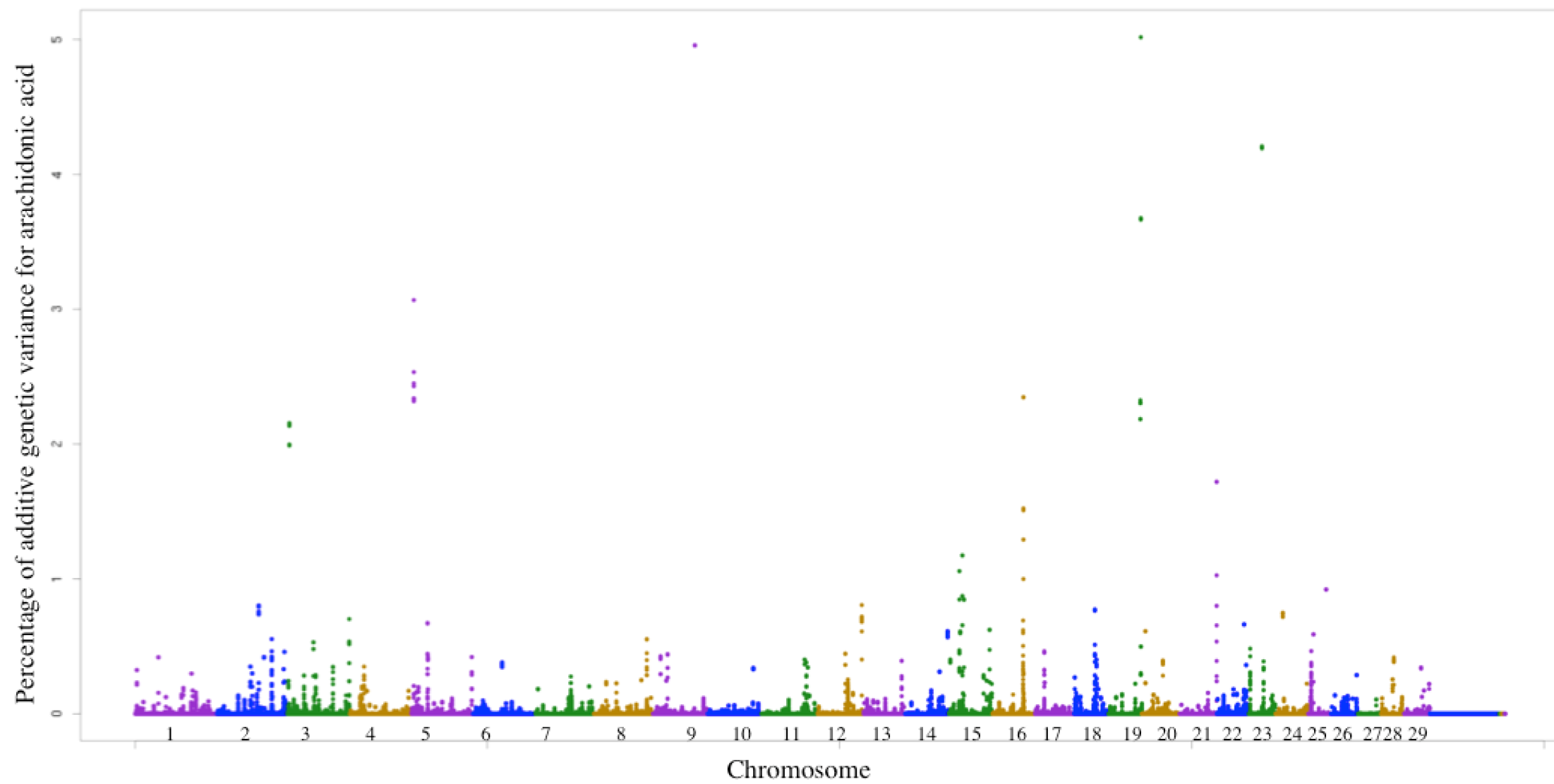

Supplement: Additional file 1: — Manhattan plot of the genome-wide association study for fatty acids in Nellore. The X-axis represents the chromosomes, and the Y-axis shows the proportion of genetic variance explained by windows of 10 adjacent SNPs in the following 18 fatty acids: arachidonic, CLA-cis, CLA-trans, docosahexaenoic, eicosatrienoic,myristoleic, MUFA, PUFA, myristic, n6:n3, oleic, omega-3, palmitic, stearic, palmitoleic and PUFA:SFA ratio in Nellore. (ZIP 1395 kb) [file 12864_2016_2511_MOESM1_ESM.zip › add/arachidonic.pdf]

Percentage of additive genetic variance for docosahexaenoic acid

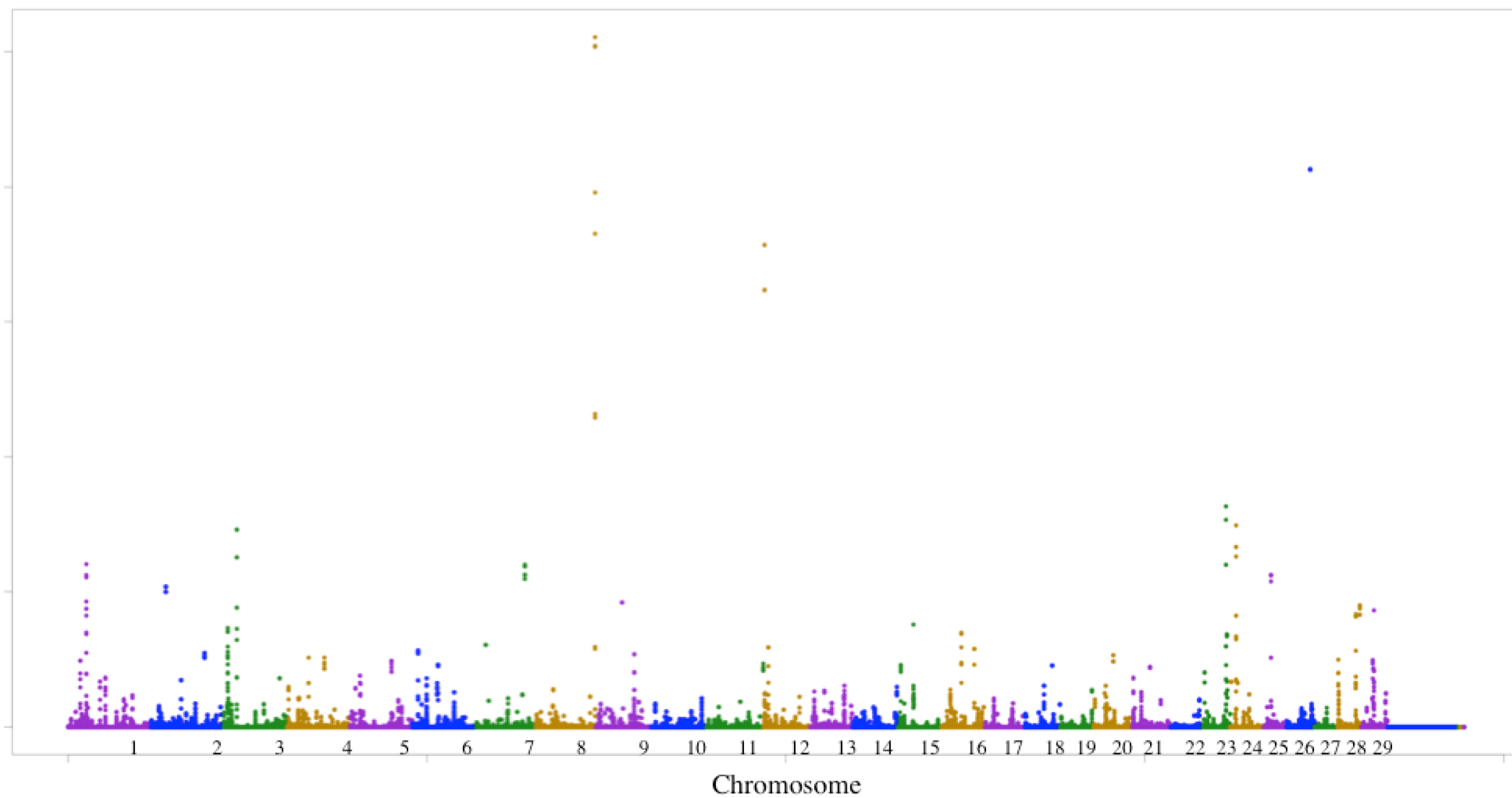

Supplement: Additional file 1: — Manhattan plot of the genome-wide association study for fatty acids in Nellore. The X-axis represents the chromosomes, and the Y-axis shows the proportion of genetic variance explained by windows of 10 adjacent SNPs in the following 18 fatty acids: arachidonic, CLA-cis, CLA-trans, docosahexaenoic, eicosatrienoic,myristoleic, MUFA, PUFA, myristic, n6:n3, oleic, omega-3, palmitic, stearic, palmitoleic and PUFA:SFA ratio in Nellore. (ZIP 1395 kb) [file 12864_2016_2511_MOESM1_ESM.zip › add/Doco.pdf]

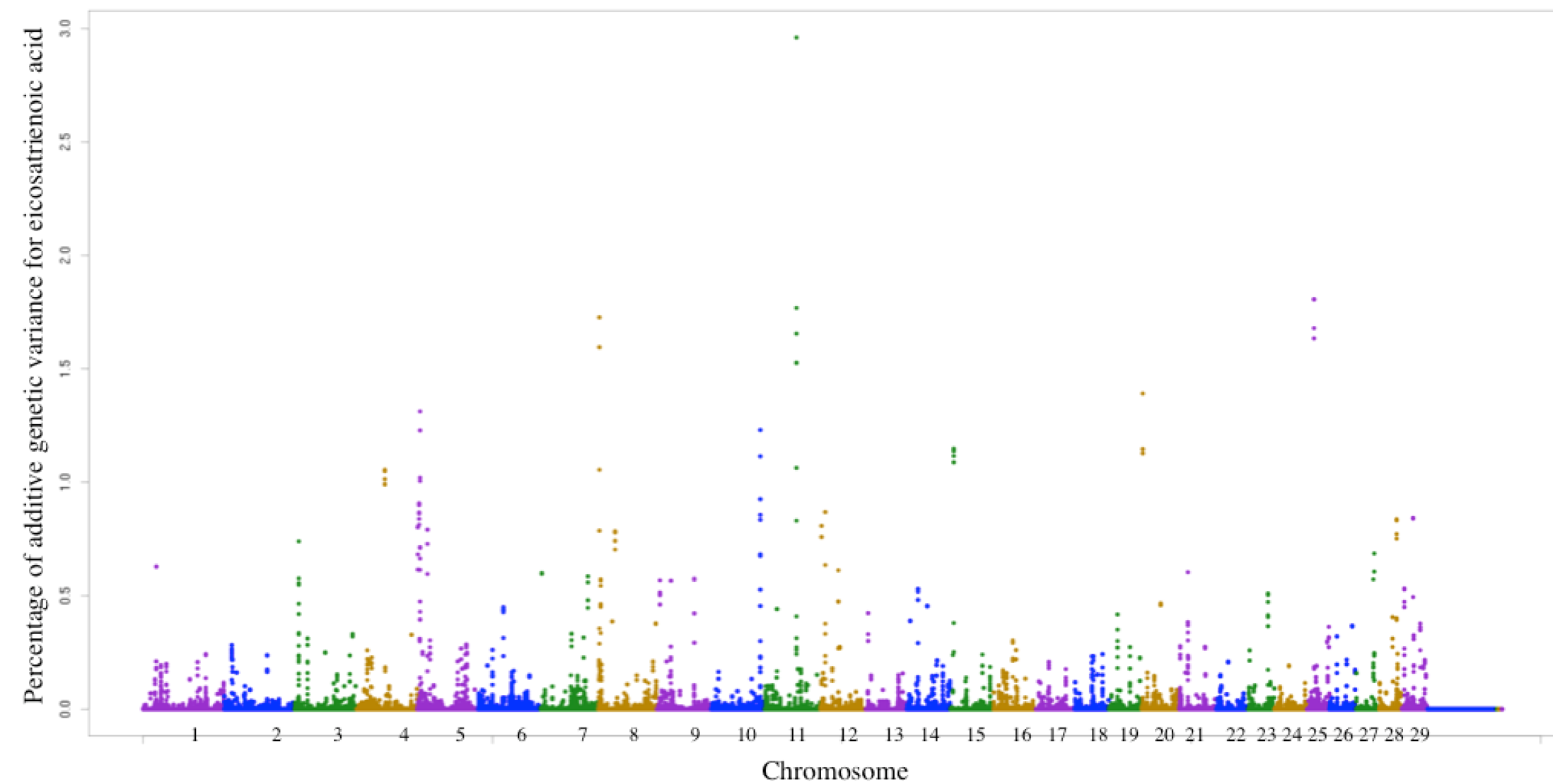

Supplement: Additional file 1: — Manhattan plot of the genome-wide association study for fatty acids in Nellore. The X-axis represents the chromosomes, and the Y-axis shows the proportion of genetic variance explained by windows of 10 adjacent SNPs in the following 18 fatty acids: arachidonic, CLA-cis, CLA-trans, docosahexaenoic, eicosatrienoic,myristoleic, MUFA, PUFA, myristic, n6:n3, oleic, omega-3, palmitic, stearic, palmitoleic and PUFA:SFA ratio in Nellore. (ZIP 1395 kb) [file 12864_2016_2511_MOESM1_ESM.zip › add/eicosatrienoic.pdf]

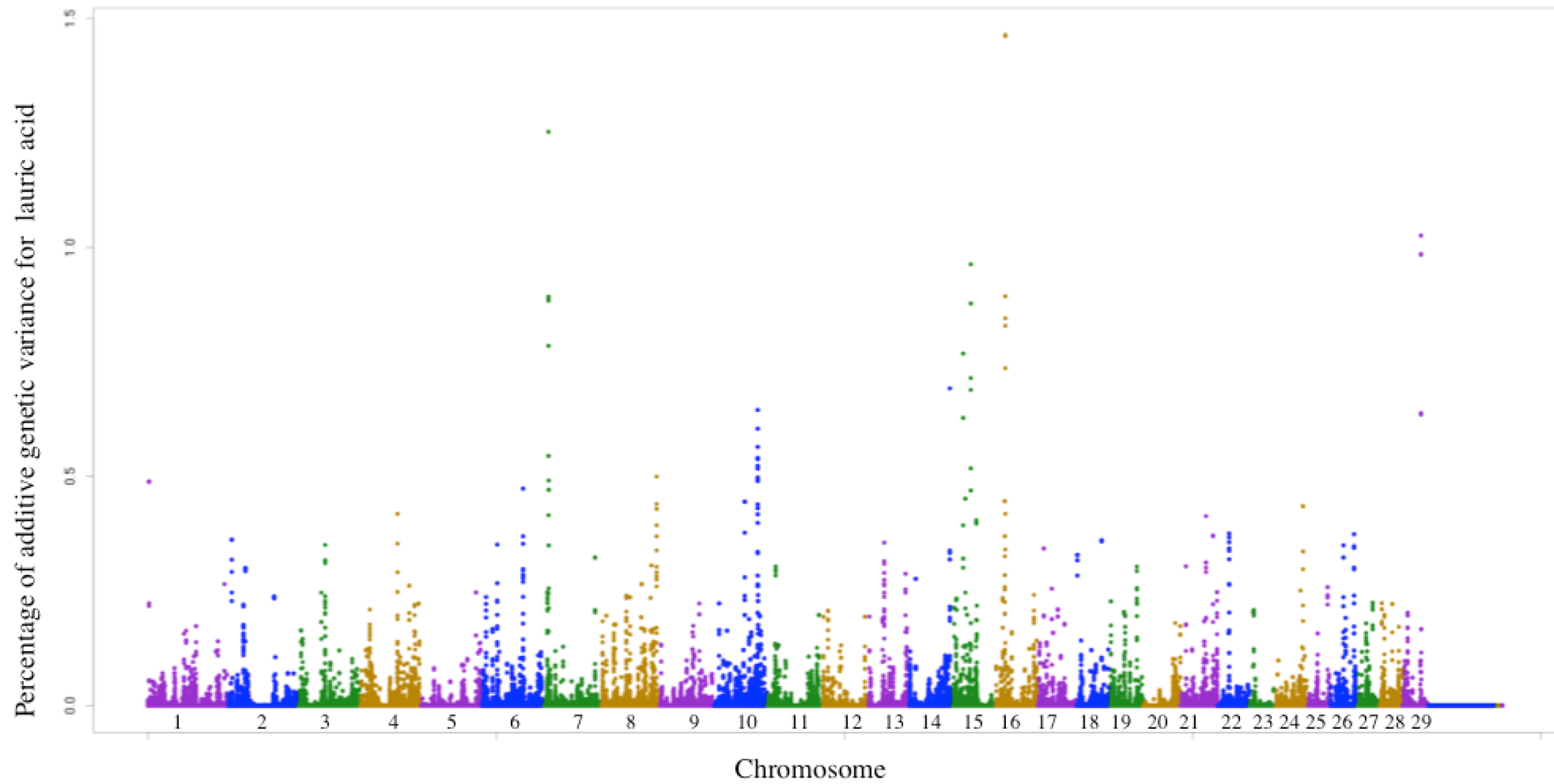

Supplement: Additional file 1: — Manhattan plot of the genome-wide association study for fatty acids in Nellore. The X-axis represents the chromosomes, and the Y-axis shows the proportion of genetic variance explained by windows of 10 adjacent SNPs in the following 18 fatty acids: arachidonic, CLA-cis, CLA-trans, docosahexaenoic, eicosatrienoic,myristoleic, MUFA, PUFA, myristic, n6:n3, oleic, omega-3, palmitic, stearic, palmitoleic and PUFA:SFA ratio in Nellore. (ZIP 1395 kb) [file 12864_2016_2511_MOESM1_ESM.zip › add/lauric.pdf]

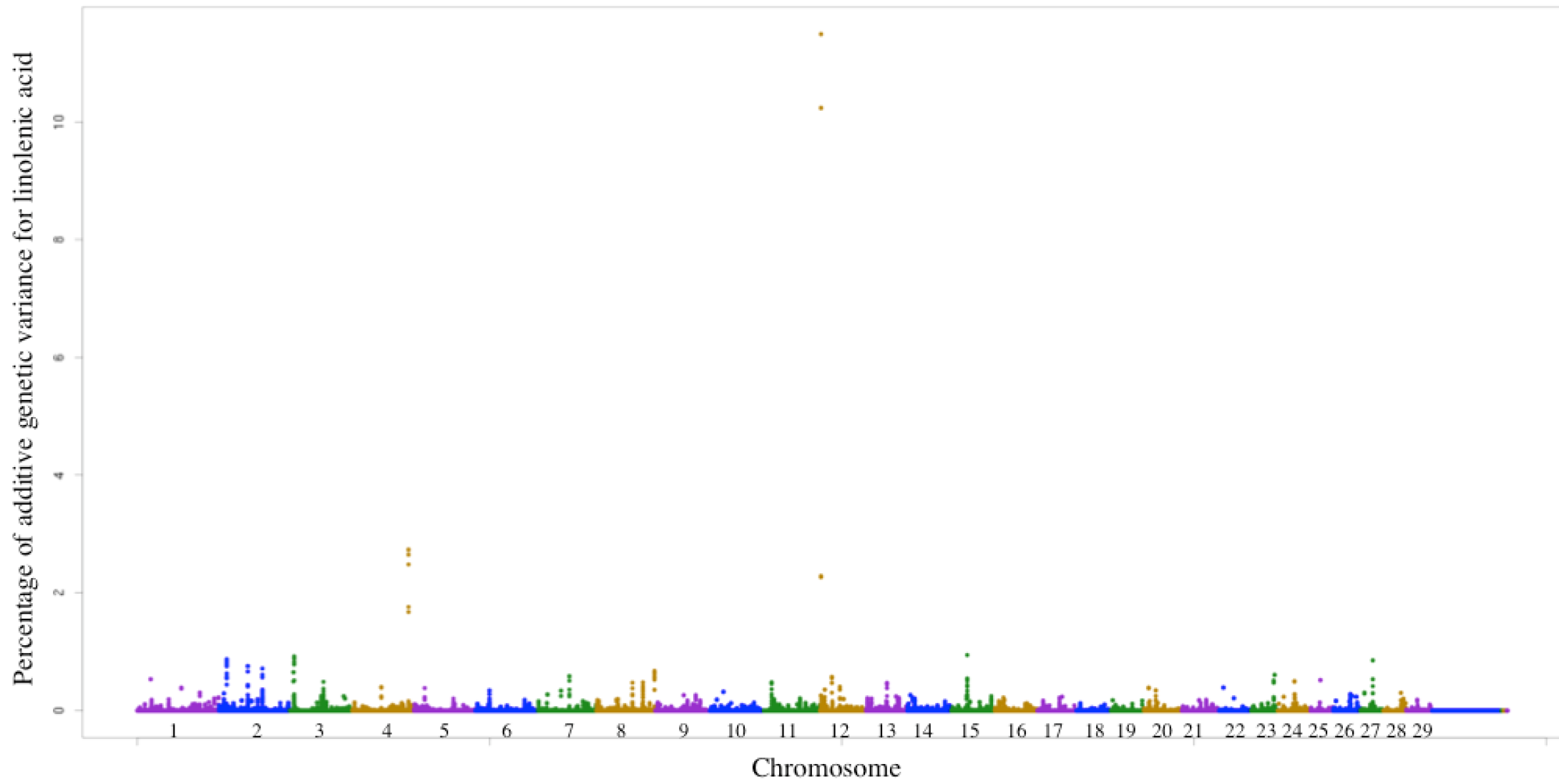

Supplement: Additional file 1: — Manhattan plot of the genome-wide association study for fatty acids in Nellore. The X-axis represents the chromosomes, and the Y-axis shows the proportion of genetic variance explained by windows of 10 adjacent SNPs in the following 18 fatty acids: arachidonic, CLA-cis, CLA-trans, docosahexaenoic, eicosatrienoic,myristoleic, MUFA, PUFA, myristic, n6:n3, oleic, omega-3, palmitic, stearic, palmitoleic and PUFA:SFA ratio in Nellore. (ZIP 1395 kb) [file 12864_2016_2511_MOESM1_ESM.zip › add/linolenic.pdf]

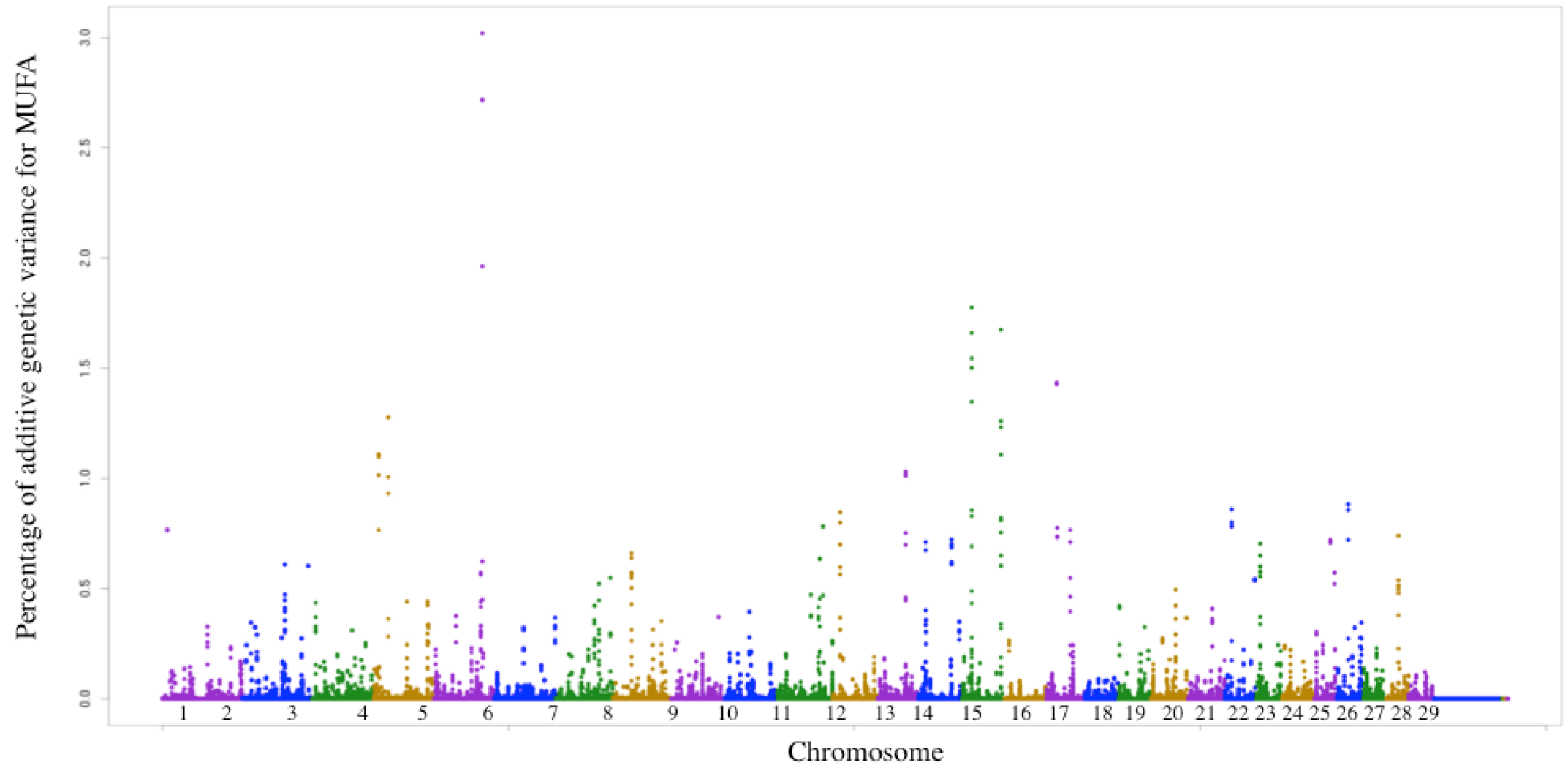

Supplement: Additional file 1: — Manhattan plot of the genome-wide association study for fatty acids in Nellore. The X-axis represents the chromosomes, and the Y-axis shows the proportion of genetic variance explained by windows of 10 adjacent SNPs in the following 18 fatty acids: arachidonic, CLA-cis, CLA-trans, docosahexaenoic, eicosatrienoic,myristoleic, MUFA, PUFA, myristic, n6:n3, oleic, omega-3, palmitic, stearic, palmitoleic and PUFA:SFA ratio in Nellore. (ZIP 1395 kb) [file 12864_2016_2511_MOESM1_ESM.zip › add/MUFA.pdf]

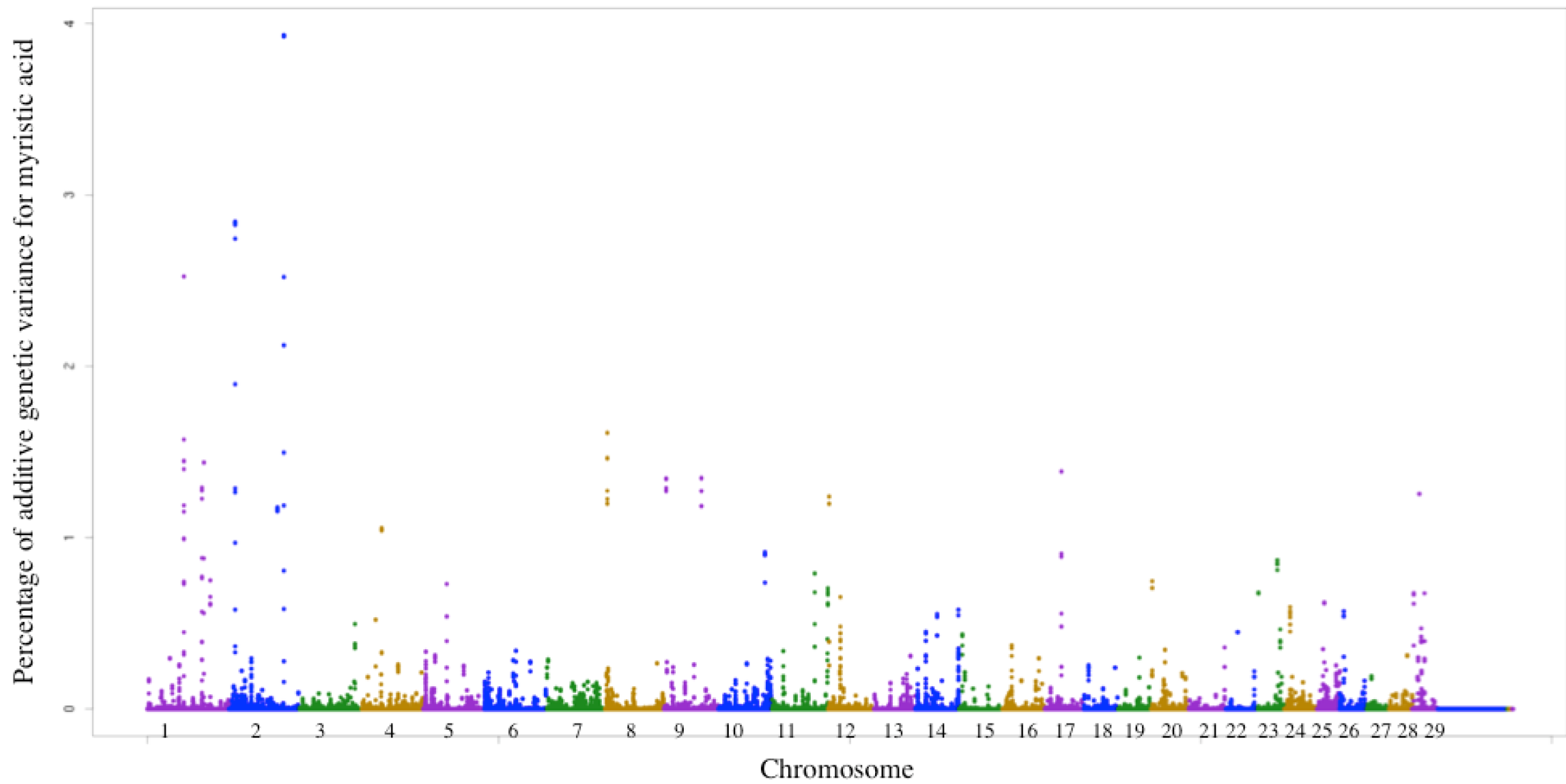

Supplement: Additional file 1: — Manhattan plot of the genome-wide association study for fatty acids in Nellore. The X-axis represents the chromosomes, and the Y-axis shows the proportion of genetic variance explained by windows of 10 adjacent SNPs in the following 18 fatty acids: arachidonic, CLA-cis, CLA-trans, docosahexaenoic, eicosatrienoic,myristoleic, MUFA, PUFA, myristic, n6:n3, oleic, omega-3, palmitic, stearic, palmitoleic and PUFA:SFA ratio in Nellore. (ZIP 1395 kb) [file 12864_2016_2511_MOESM1_ESM.zip › add/Myristic.pdf]

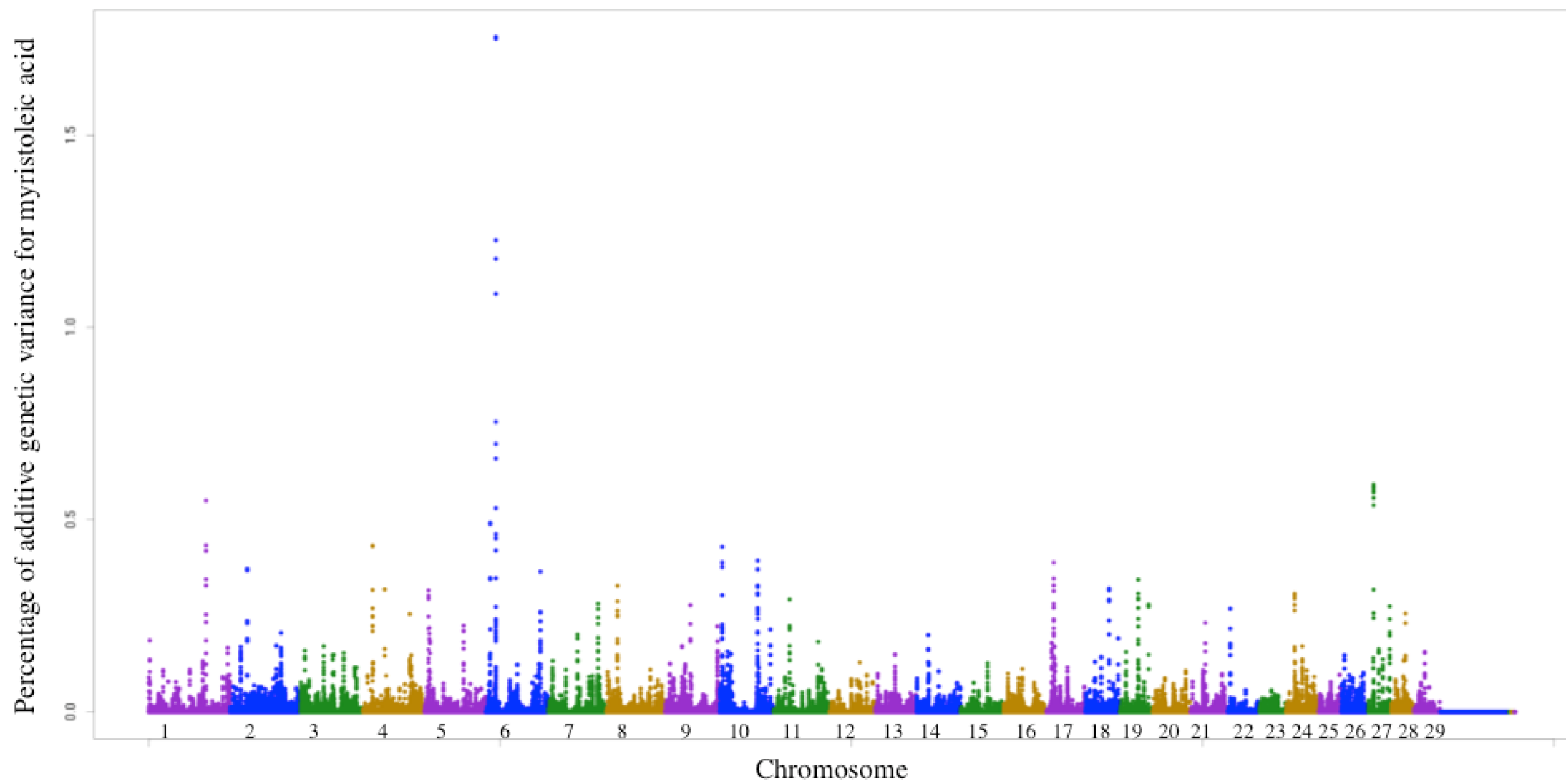

Supplement: Additional file 1: — Manhattan plot of the genome-wide association study for fatty acids in Nellore. The X-axis represents the chromosomes, and the Y-axis shows the proportion of genetic variance explained by windows of 10 adjacent SNPs in the following 18 fatty acids: arachidonic, CLA-cis, CLA-trans, docosahexaenoic, eicosatrienoic,myristoleic, MUFA, PUFA, myristic, n6:n3, oleic, omega-3, palmitic, stearic, palmitoleic and PUFA:SFA ratio in Nellore. (ZIP 1395 kb) [file 12864_2016_2511_MOESM1_ESM.zip › add/Myristoleic.pdf]

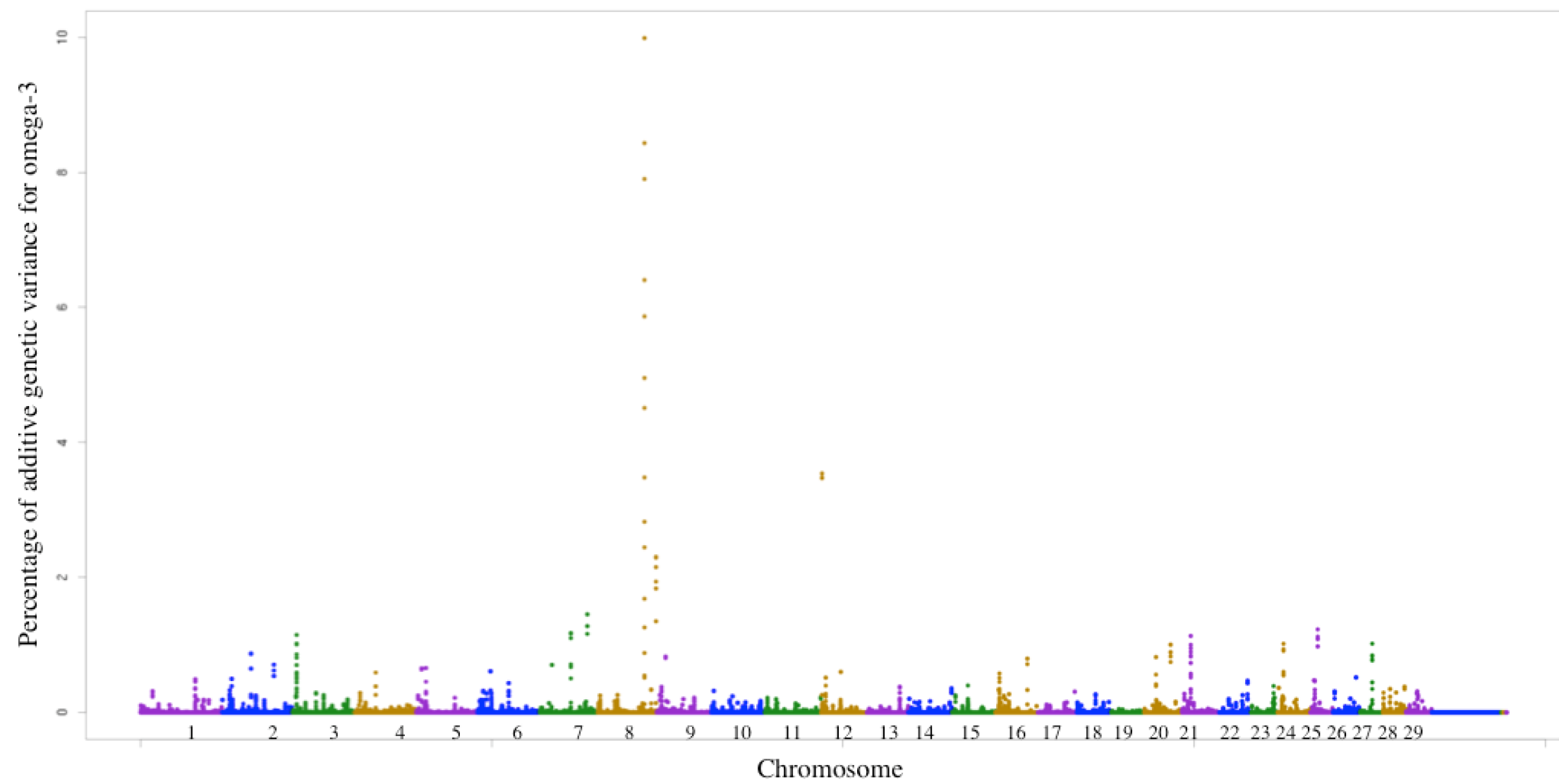

Supplement: Additional file 1: — Manhattan plot of the genome-wide association study for fatty acids in Nellore. The X-axis represents the chromosomes, and the Y-axis shows the proportion of genetic variance explained by windows of 10 adjacent SNPs in the following 18 fatty acids: arachidonic, CLA-cis, CLA-trans, docosahexaenoic, eicosatrienoic,myristoleic, MUFA, PUFA, myristic, n6:n3, oleic, omega-3, palmitic, stearic, palmitoleic and PUFA:SFA ratio in Nellore. (ZIP 1395 kb) [file 12864_2016_2511_MOESM1_ESM.zip › add/n3.pdf]

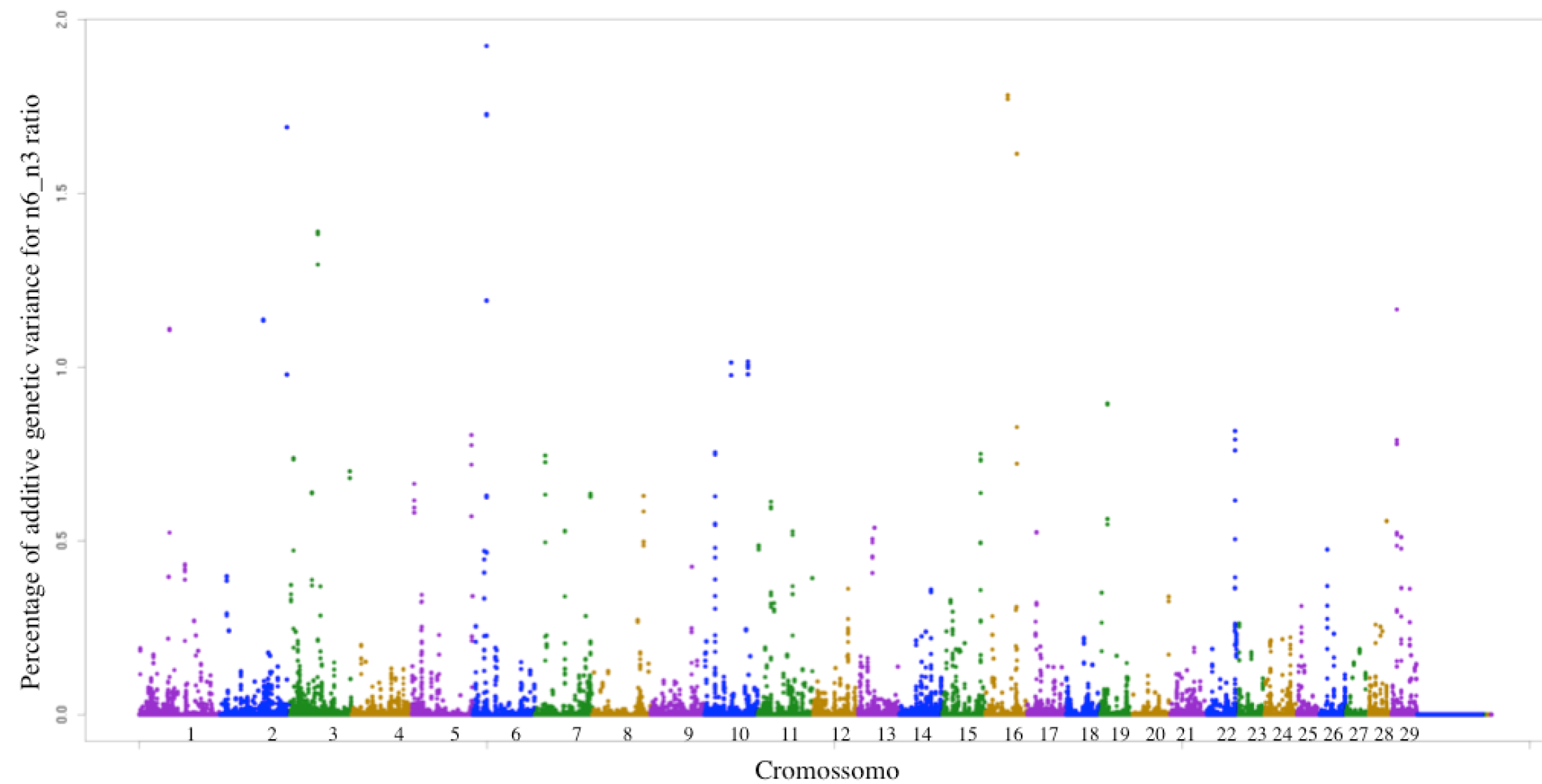

Supplement: Additional file 1: — Manhattan plot of the genome-wide association study for fatty acids in Nellore. The X-axis represents the chromosomes, and the Y-axis shows the proportion of genetic variance explained by windows of 10 adjacent SNPs in the following 18 fatty acids: arachidonic, CLA-cis, CLA-trans, docosahexaenoic, eicosatrienoic,myristoleic, MUFA, PUFA, myristic, n6:n3, oleic, omega-3, palmitic, stearic, palmitoleic and PUFA:SFA ratio in Nellore. (ZIP 1395 kb) [file 12864_2016_2511_MOESM1_ESM.zip › add/n6n3.pdf]

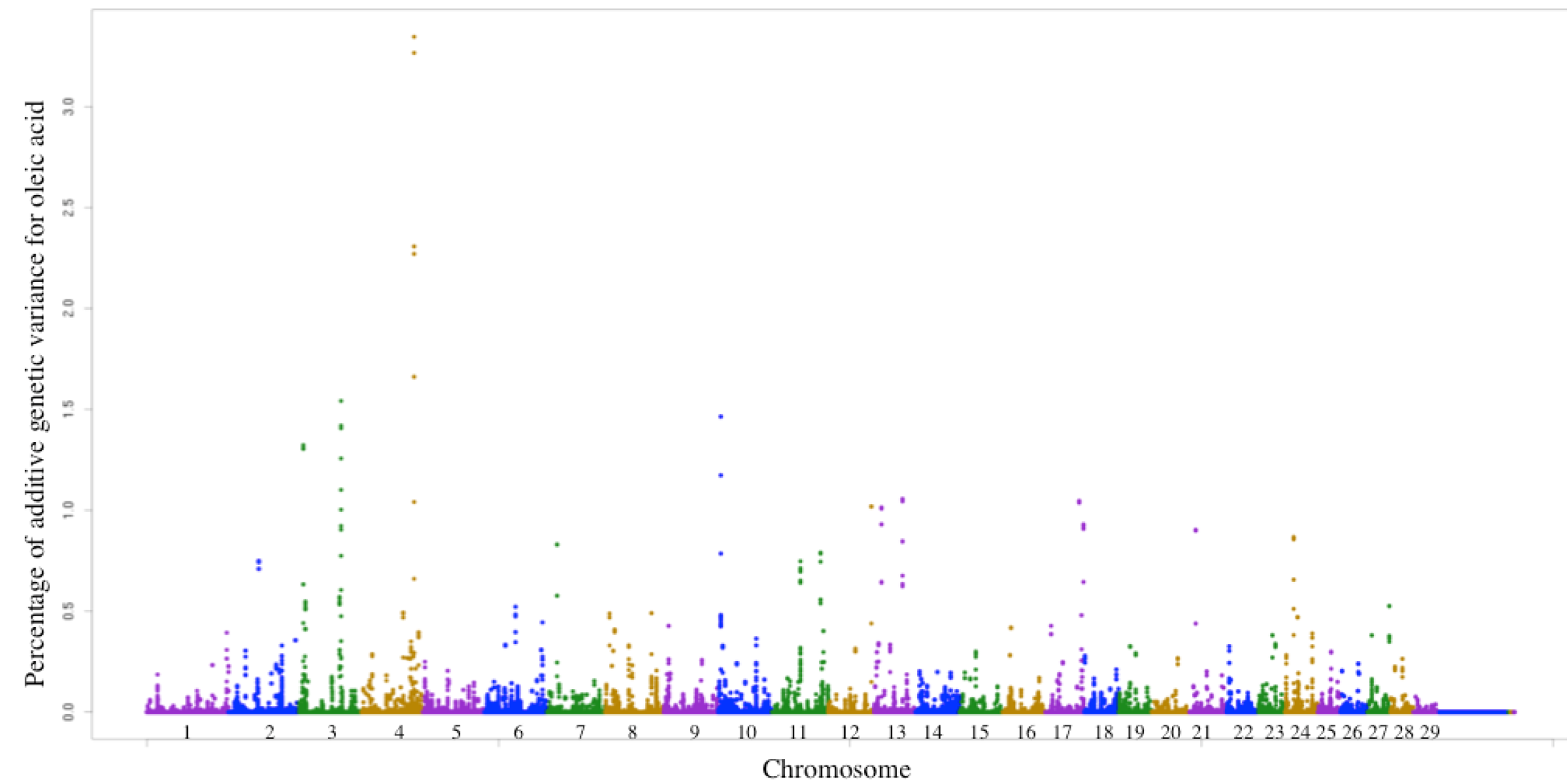

Supplement: Additional file 1: — Manhattan plot of the genome-wide association study for fatty acids in Nellore. The X-axis represents the chromosomes, and the Y-axis shows the proportion of genetic variance explained by windows of 10 adjacent SNPs in the following 18 fatty acids: arachidonic, CLA-cis, CLA-trans, docosahexaenoic, eicosatrienoic,myristoleic, MUFA, PUFA, myristic, n6:n3, oleic, omega-3, palmitic, stearic, palmitoleic and PUFA:SFA ratio in Nellore. (ZIP 1395 kb) [file 12864_2016_2511_MOESM1_ESM.zip › add/oleic.pdf]

Percentage of additive genetic variance for palmitic acid

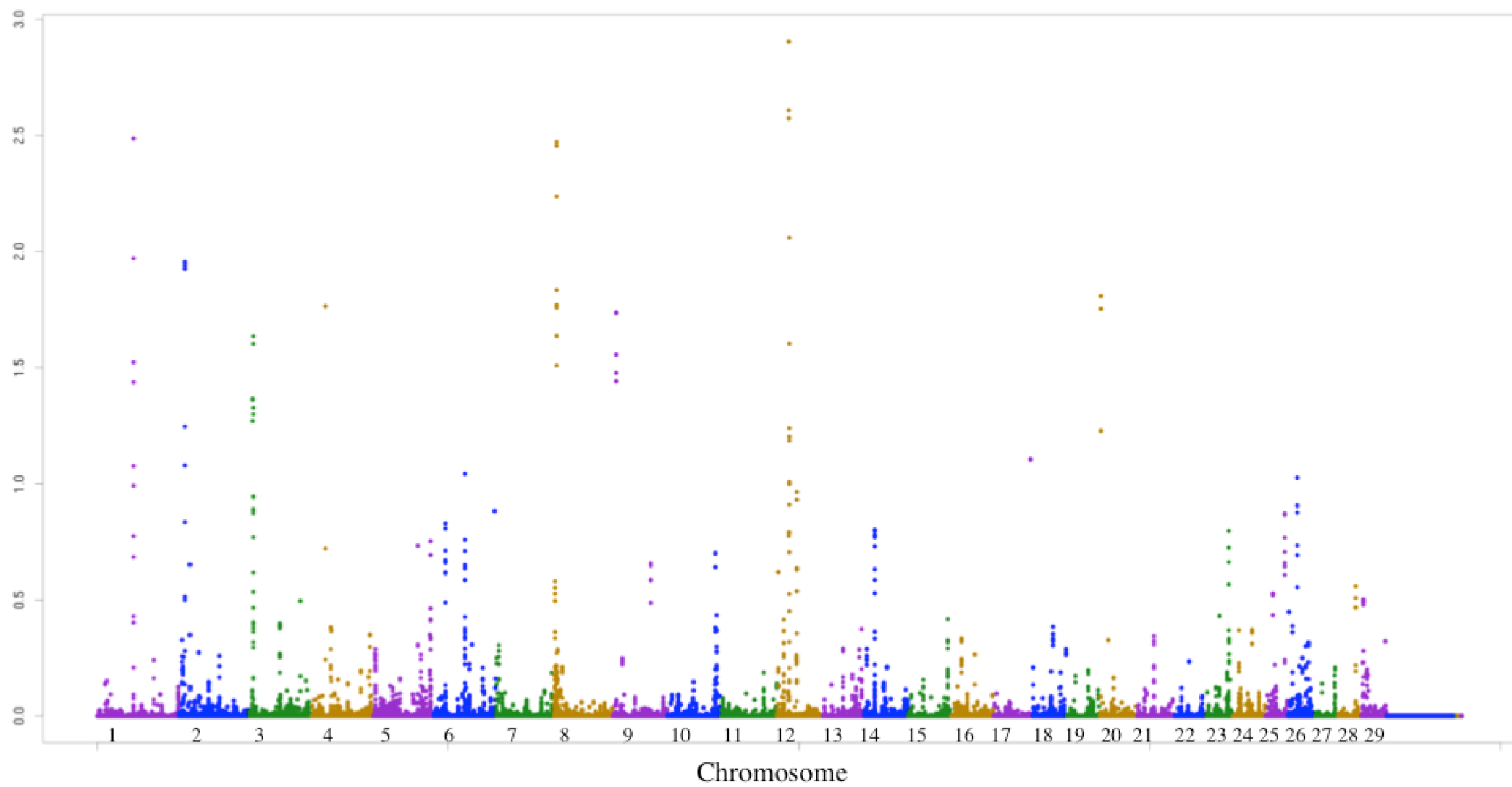

Supplement: Additional file 1: — Manhattan plot of the genome-wide association study for fatty acids in Nellore. The X-axis represents the chromosomes, and the Y-axis shows the proportion of genetic variance explained by windows of 10 adjacent SNPs in the following 18 fatty acids: arachidonic, CLA-cis, CLA-trans, docosahexaenoic, eicosatrienoic,myristoleic, MUFA, PUFA, myristic, n6:n3, oleic, omega-3, palmitic, stearic, palmitoleic and PUFA:SFA ratio in Nellore. (ZIP 1395 kb) [file 12864_2016_2511_MOESM1_ESM.zip › add/palmitic.pdf]

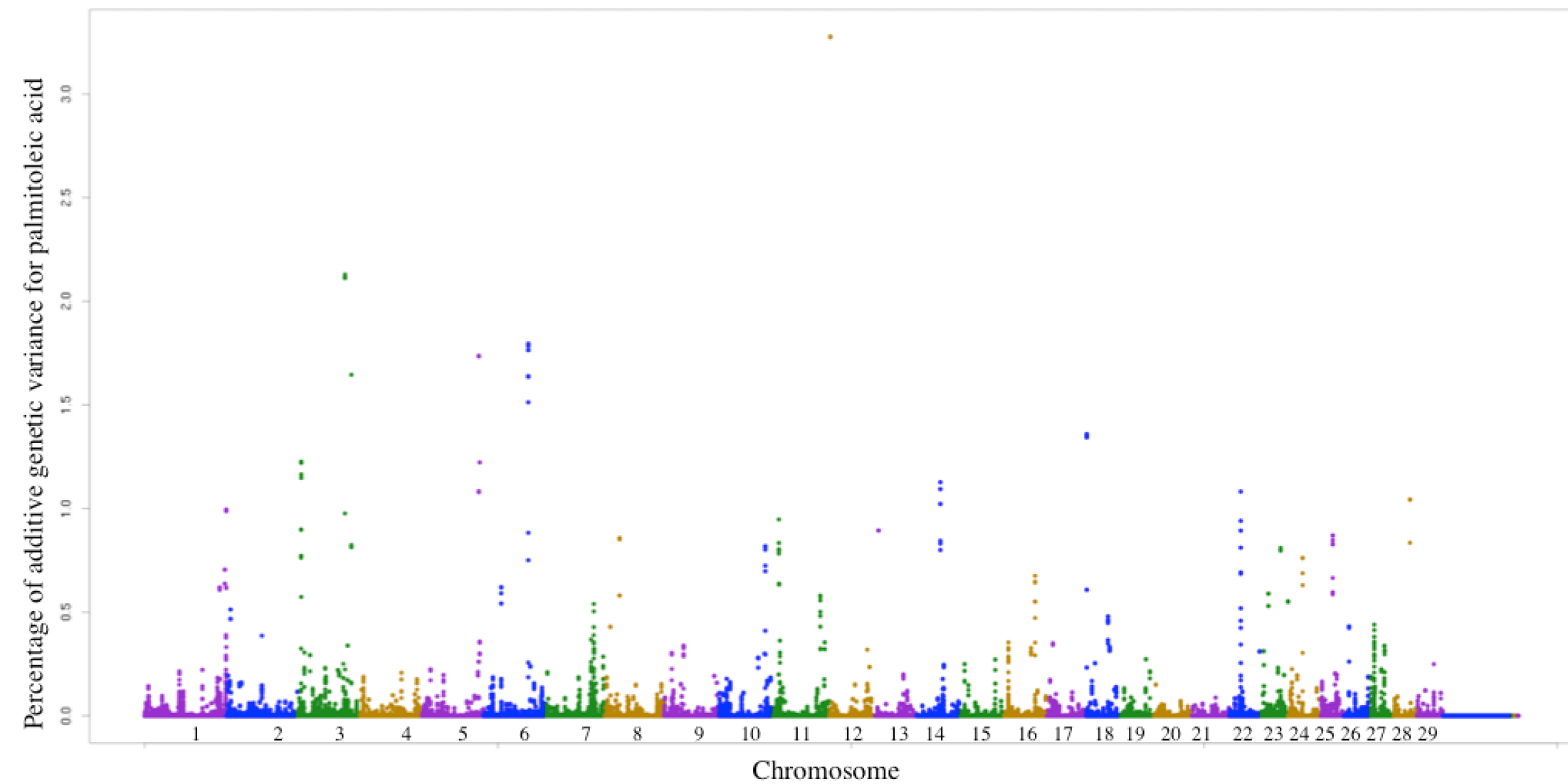

Supplement: Additional file 1: — Manhattan plot of the genome-wide association study for fatty acids in Nellore. The X-axis represents the chromosomes, and the Y-axis shows the proportion of genetic variance explained by windows of 10 adjacent SNPs in the following 18 fatty acids: arachidonic, CLA-cis, CLA-trans, docosahexaenoic, eicosatrienoic,myristoleic, MUFA, PUFA, myristic, n6:n3, oleic, omega-3, palmitic, stearic, palmitoleic and PUFA:SFA ratio in Nellore. (ZIP 1395 kb) [file 12864_2016_2511_MOESM1_ESM.zip › add/palmitoleic.pdf]

Percentage of additive genetic variance for PUFA

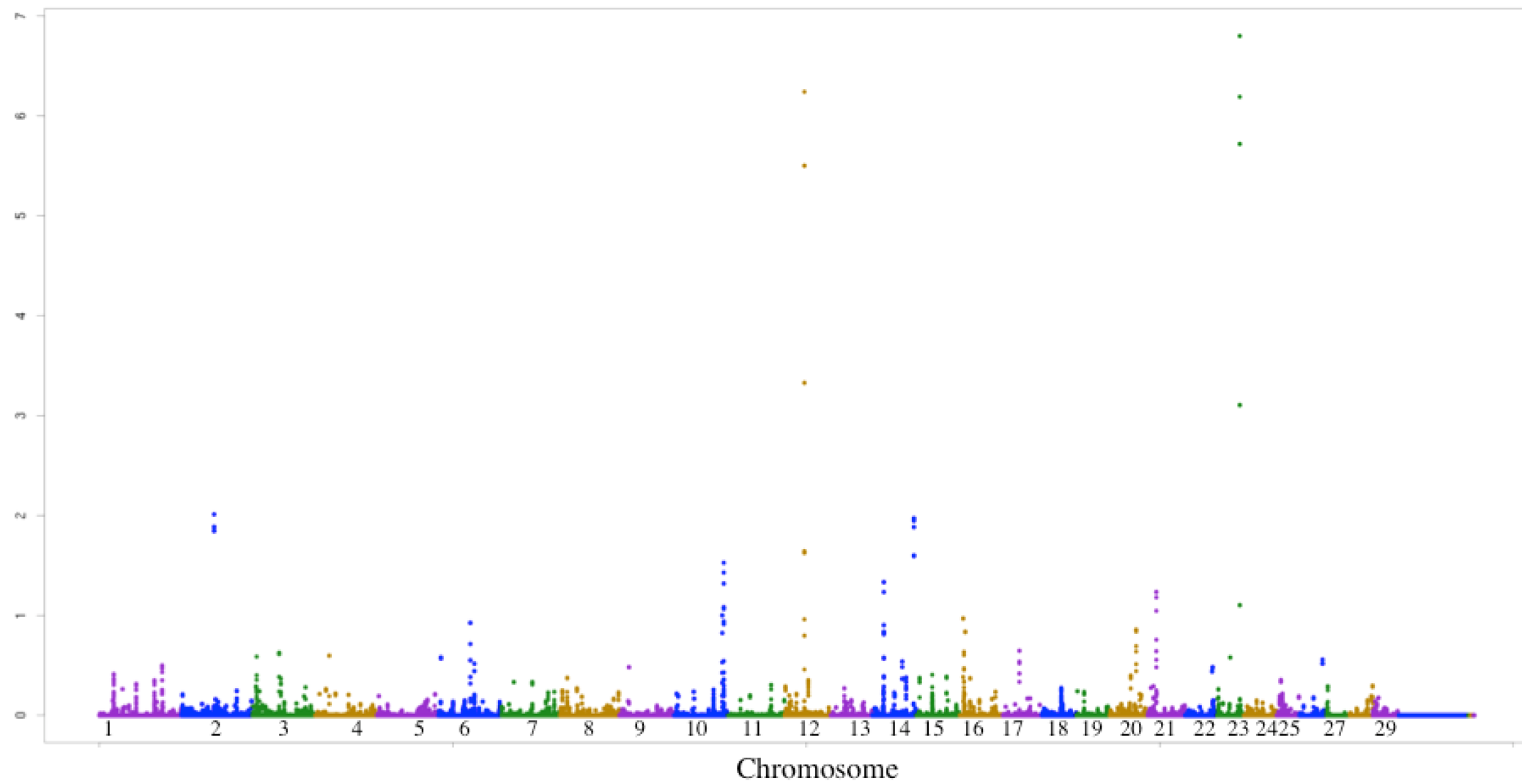

Supplement: Additional file 1: — Manhattan plot of the genome-wide association study for fatty acids in Nellore. The X-axis represents the chromosomes, and the Y-axis shows the proportion of genetic variance explained by windows of 10 adjacent SNPs in the following 18 fatty acids: arachidonic, CLA-cis, CLA-trans, docosahexaenoic, eicosatrienoic,myristoleic, MUFA, PUFA, myristic, n6:n3, oleic, omega-3, palmitic, stearic, palmitoleic and PUFA:SFA ratio in Nellore. (ZIP 1395 kb) [file 12864_2016_2511_MOESM1_ESM.zip › add/PUFA.pdf]

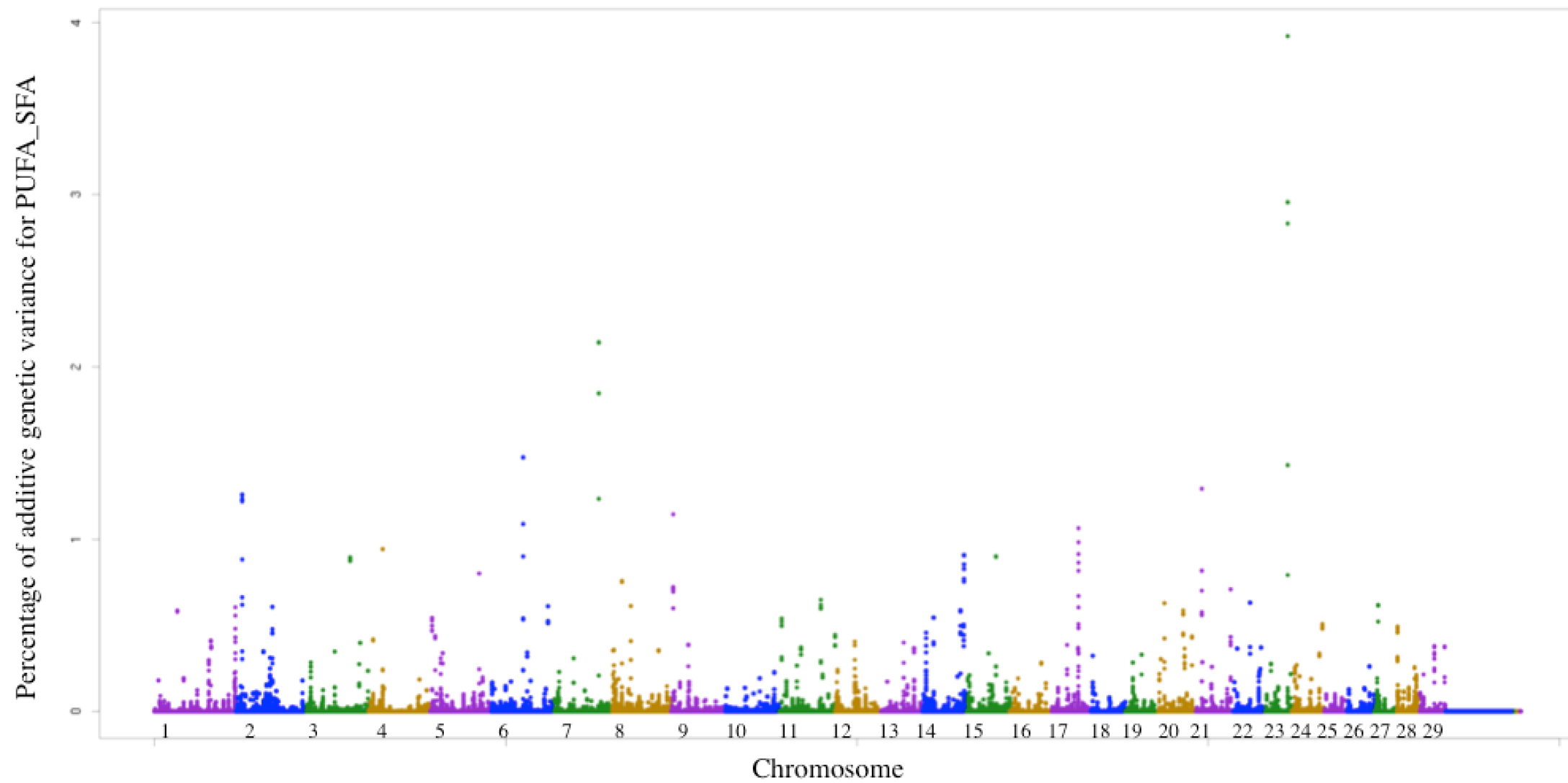

Supplement: Additional file 1: — Manhattan plot of the genome-wide association study for fatty acids in Nellore. The X-axis represents the chromosomes, and the Y-axis shows the proportion of genetic variance explained by windows of 10 adjacent SNPs in the following 18 fatty acids: arachidonic, CLA-cis, CLA-trans, docosahexaenoic, eicosatrienoic,myristoleic, MUFA, PUFA, myristic, n6:n3, oleic, omega-3, palmitic, stearic, palmitoleic and PUFA:SFA ratio in Nellore. (ZIP 1395 kb) [file 12864_2016_2511_MOESM1_ESM.zip › add/PUFA_SFA.pdf]

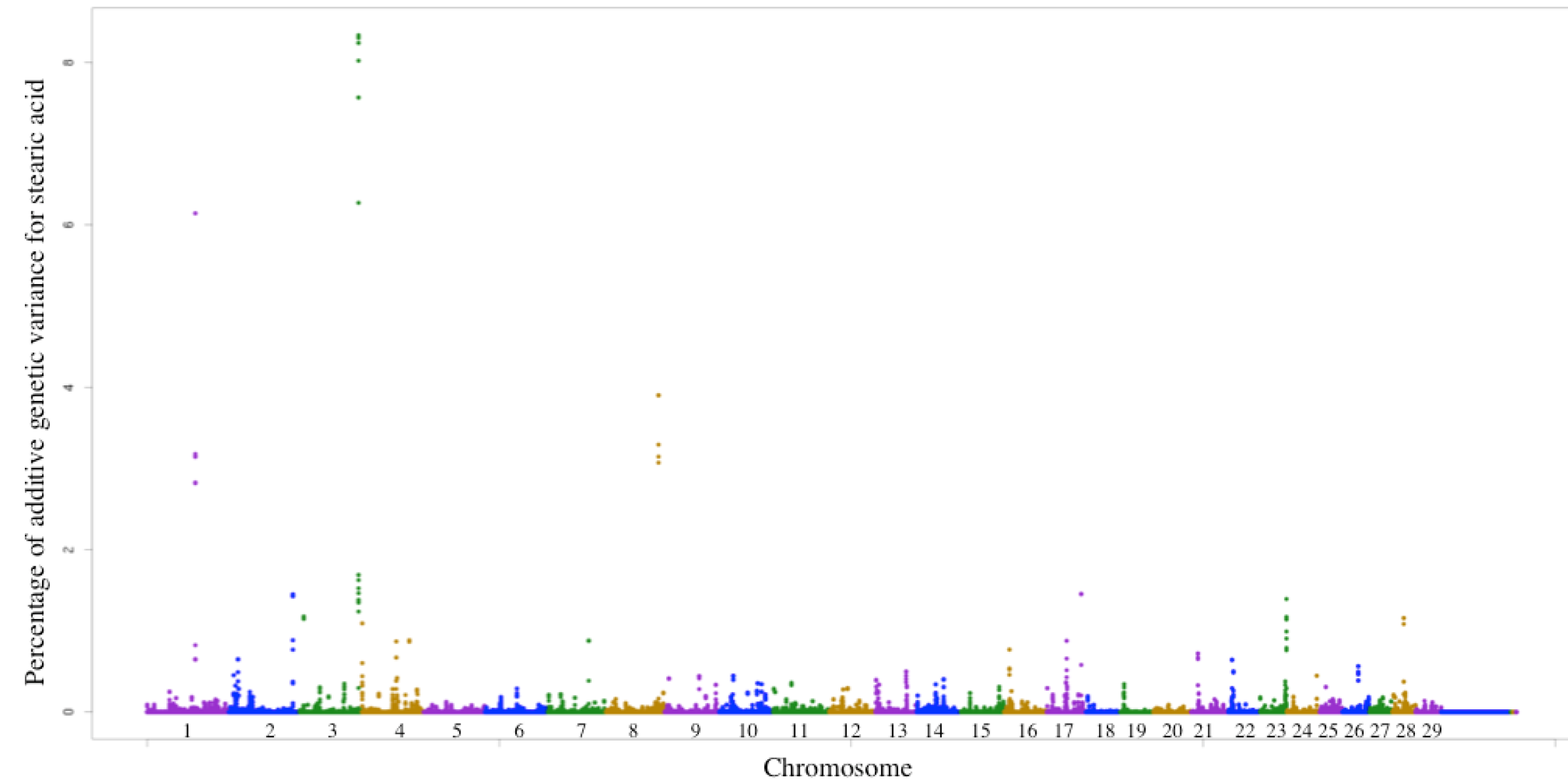

Supplement: Additional file 1: — Manhattan plot of the genome-wide association study for fatty acids in Nellore. The X-axis represents the chromosomes, and the Y-axis shows the proportion of genetic variance explained by windows of 10 adjacent SNPs in the following 18 fatty acids: arachidonic, CLA-cis, CLA-trans, docosahexaenoic, eicosatrienoic,myristoleic, MUFA, PUFA, myristic, n6:n3, oleic, omega-3, palmitic, stearic, palmitoleic and PUFA:SFA ratio in Nellore. (ZIP 1395 kb) [file 12864_2016_2511_MOESM1_ESM.zip › add/stearic.pdf]
